# Respiratory Microbiome Profiling for Etiologic Diagnosis of Pneumonia in Mechanically Ventilated Patients

**DOI:** 10.3389/fmicb.2018.01413

**Published:** 2018-07-10

**Authors:** Georgios D. Kitsios, Adam Fitch, Dimitris V. Manatakis, Sarah F. Rapport, Kelvin Li, Shulin Qin, Joseph Huwe, Yingze Zhang, Yohei Doi, John Evankovich, William Bain, Janet S. Lee, Barbara Methé, Panayiotis V. Benos, Alison Morris, Bryan J. McVerry

**Affiliations:** ^1^Division of Pulmonary, Allergy and Critical Care Medicine, Department of Medicine, University of Pittsburgh School of Medicine and University of Pittsburgh Medical Center, Pittsburgh, PA, United States; ^2^Center for Medicine and the Microbiome, University of Pittsburgh, Pittsburgh, PA, United States; ^3^Department of Computational and Systems Biology, University of Pittsburgh, Pittsburgh, PA, United States; ^4^Division of Infectious Diseases, University of Pittsburgh Medical Center, Pittsburgh, PA, United States; ^5^Department of Immunology, University of Pittsburgh School of Medicine, Pittsburgh, PA, United States

**Keywords:** microbiome, pneumonia, 16S rRNA gene sequencing, respiratory failure, antibiotic stewardship

## Abstract

Etiologic diagnosis of bacterial pneumonia relies on identification of causative pathogens by cultures, which require extended incubation periods and have limited sensitivity. Next-generation sequencing of microbial DNA directly from patient samples may improve diagnostic accuracy for guiding antibiotic prescriptions. In this study, we hypothesized that enhanced pathogen detection using sequencing can improve upon culture-based diagnosis and that certain sequencing profiles correlate with host response. We prospectively collected endotracheal aspirates and plasma within 72 h of intubation from patients with acute respiratory failure. We performed 16S rRNA gene sequencing to determine pathogen abundance in lung samples and measured plasma biomarkers to assess host responses to detected pathogens. Among 56 patients, 12 patients (21%) had positive respiratory cultures. Sequencing revealed lung communities with low diversity (*p* < 0.02) dominated by taxa (>50% relative abundance) corresponding to clinically isolated pathogens (concordance *p* = 0.009). Importantly, sequencing detected dominant pathogens in 20% of the culture-negative patients exposed to broad-spectrum empiric antibiotics. Regardless of culture results, pathogen dominance correlated with increased plasma markers of host injury (receptor of advanced glycation end-products-RAGE) and inflammation (interleukin-6, tumor necrosis factor receptor 1-TNFR1) (*p* < 0.05), compared to subjects without dominant pathogens in their lung communities. Machine-learning algorithms identified pathogen abundance by sequencing as the most informative predictor of culture positivity. Thus, enhanced detection of pathogenic bacteria by sequencing improves etiologic diagnosis of pneumonia, correlates with host responses, and offers substantial opportunity for individualized therapeutic targeting and antimicrobial stewardship. Clinical translation will require validation with rapid whole meta-genome sequencing approaches to guide real-time antibiotic prescriptions.

## Introduction

Severe pneumonia is a leading cause of hospitalization and death among adults in the US, often requiring admission to an intensive care unit (ICU) (Chalmers et al., 2011; Barrett et al., 2014; Jain et al., 2015; Valley et al., 2015). While appropriate antibiotic therapy is the cornerstone of pneumonia management, etiologic pathogen diagnosis with current culture-based microbiologic tests is often negative in patients with a clinical picture of pneumonia (Jain et al., 2015) or requires long incubation periods (∼3 days) to provide actionable results (Zumla et al., 2014; Jain et al., 2015; Vincent et al., 2015). Consequently, antibiotic prescriptions for severe pneumonia are empiric and typically include two or three broad-spectrum agents prescribed for seven or more days (Mandell et al., 2007; Kalil et al., 2016). This “one-size-fits-all” practice is hazardous for individual patients, who may receive insufficient or disproportionately intense antibiotics, and further contributes to antibiotic resistance, a global health threat (Laxminarayan et al., 2013; Modi et al., 2014; Kitsios et al., 2017).

Next-generation sequencing (NGS) of microbial DNA extracted directly from patient samples without the need for *ex vivo* organismal growth may overcome shortcomings of culture-based diagnosis. By sequencing either amplified bacterial marker genes (typically the 16S rRNA gene [16S sequencing]) or whole metagenomes, NGS provides comprehensive profiling of resident microbial communities with relative abundance information of the constituent bacteria, regardless of whether they are alive, dead or fastidious. With improvements in fidelity and accessibility of whole metagenome sequencing, identification of non-bacterial microbes (viruses, fungi and parasites) (Kuczynski et al., 2011) is becoming increasingly feasible and holds promise for clinical utility in the near future (Judge et al., 2015). Despite the theoretical advantages of NGS, the technology has not yet been validated as a diagnostic tool to guide antimicrobial prescriptions in the ICU.

To examine the clinical validity (Khoury et al., 2003) of 16S sequencing for etiologic diagnosis of bacterial pneumonia in patients requiring mechanical ventilation, we conducted this proof-of-concept study with the Microbiome Cohort in Acute Lung Injury Registry (MICALIR) at the University of Pittsburgh Medical Center (UPMC), assessing the upper and lower respiratory tract microbiome composition and its association with clinical diagnoses, host responses, and clinical outcomes.

## Materials and Methods

This study is reported in compliance with the Strengthening the Reporting of Observational studies in Epidemiology (STROBE) statement (details presented in Supplementary Table 1) (Elm et al., 2007). Extensive methods regarding clinical data recording, sample collection, experimental protocols and statistical analyses are presented in the Supplement. Primary data and statistical code for replication of our findings are also provided in the online Supplement and are also available for download at https://github.com/MicrobiomeALIR/Resp_Microbiome_Profiles_Pneumonia.

### Study Design and Participants

We conducted a prospective cohort study from June 2015 – March 2017 enrolling consecutive adult patients in the Medical ICU with acute respiratory failure within 72 h of intubation. Eligible patients were 18 years or older with acute respiratory failure requiring mechanical ventilation via endotracheal intubation. Exclusion criteria included inability to obtain informed consent, presence of tracheostomy, or mechanical ventilation for more than 72 h. Given our focus on etiologic diagnosis of bacterial pneumonia, we divided patients into culture-positive and culture-negative cases, based on clinical microbiologic results of respiratory specimens. We considered specimens obtained within 48 h of research sample timing acquisition so that such specimens would be reflective of the same infectious process being studied by NGS. Clinical cultures were obtained at the discretion of the treating physicians who were not involved in the MICALIR study. We considered microbiologic cultures of respiratory specimens [sputum, endo-tracheal aspirates (ETAs), or bronchoalveolar lavage – (BAL)] as positive when pathogenic bacterial species had been isolated by the clinical laboratory and treating physicians covered these bacteria with antibiotics (i.e., clinically these bacteria were not considered to be airway colonizers). Culture-negative cases were defined as those in which no organismal growth was observed or presence of “normal respiratory flora” was reported, as per standard clinical practices. We also recorded results of respiratory viral panel testing performed in nasopharyngeal swabs or respiratory specimens. The University of Pittsburgh Institutional Review Board approved the MICALIR study and written informed consent was provided by all participants or their surrogates in accordance with the Declaration of Helsinki.

### Clinical Data Collection

We collected prospective clinical data on participants including age, gender, body mass index and history of smoking, comorbid conditions, such as diabetes and chronic obstructive pulmonary disease (COPD), on the day of enrollment. Physiological and laboratory variables such as PaO_2_ to FiO_2_ ratio, levels of positive end-expiratory pressure, plateau pressure, systolic blood pressure, white blood cell count etc. were obtained from the medical record by recording the physiologically worse value within a 24 h period on the day of enrollment (e.g., lowest blood pressure or highest creatinine value). We measured modified sequential organ failure assessment (SOFA) scores (we did not include the neurologic component of SOFA score because all patients were intubated and receiving sedative medications) by using the physiologically worse values within 24 h of enrollment. We recorded antibiotics and vasopressors administered during the first week of ICU course from intubation. A consensus committee of clinical experts (GDK, JE, WB, JSL, AM, BJM) reviewed all available data for the enrolled patients and performed retrospective classifications of the etiology and severity of their acute respiratory failure. Classifications were performed without knowledge of microbiome sequencing or host biomarker data and included sepsis, acute respiratory distress syndrome (ARDS), pneumonia or aspiration, and intubation for airway protection without risk factors for ARDS per established criteria (Gong et al., 2005; ARDS Definition Task Force et al., 2012; Singer et al., 2016; Neto et al., 2016). Prospective clinical outcomes included 30-day mortality, duration of ICU stay, acute kidney injury (Mehta et al., 2007), incident shock (defined as need for vasopressors) and ventilator-free days (VFD) (Kalanuria et al., 2014).

### Sample Collection

Immediately after enrollment, we obtained baseline samples for microbiome analyses of the oral and lung communities, with swabs of the base of the tongue and ETA, respectively. Simultaneous blood samples (10cc) were collected for centrifugation and separation of plasma. All samples were frozen and stored at -80°C.

### Laboratory Analyses

We extracted bacterial DNA directly from oral swabs and ETAs and amplified the V4 hypervariable region of the 16S rRNA gene for sequencing on the Illumina MiSeq platform (Morris et al., 2013). We also performed qPCR of the V3-V4 region of the 16S rRNA gene to obtain absolute bacterial loads in each sample (Liu et al., 2012). For plasma biomarker analyses, we constructed a custom Luminex multi-analyte panel (R&D Systems, Minneapolis, MI, United States) (McKay et al., 2017) targeting biomarkers associated with pneumonia diagnosis (procalcitonin) and acute lung injury outcomes (RAGE: receptor of advanced glycation end-products, IL-6: interleukin-6, IL-8: interleukin-8, sTNFR1: soluble tumor necrosis factor receptor 1) (Narvaez-Rivera et al., 2012; Calfee et al., 2014; Famous et al., 2017). Clinical microbiologic cultures were processed per standard procedures as described in the Supplement.

### Data Processing and Statistical Analysis Methods

From derived 16S sequences, we applied a custom pipeline for Operational Taxonomic Units (OTUs-taxa) classification (Supplement). We calculated descriptive statistics of baseline characteristics and performed non-parametric comparisons using the R software (R Foundation for Statistical Computing, 2016). We performed ecological analyses of alpha diversity (richness-Shannon and evenness-Dominance), beta diversity (Bray–Curtis dissimilarity index), and taxonomic descriptions between culture-positive and negative cases with the Quantitative Insights in Microbial Ecology software (QIIME) and the R *vegan* package (Dixon, 2003; Caporaso et al., 2010). Beta-diversity comparisons with permutation analysis of variance (Permanova at 1000 permutations) were visualized with non-metric multidimensional scaling (NMDS). From the taxonomic composition of the reference-standard culture-positive cases and the available literature on the composition of the healthy lung microbiome (Morris et al., 2013; Segal et al., 2016), we operationally defined taxa as “pathogenic” (when corresponding to clinically relevant bacterial species isolated in cultures) vs. “oral-origin” taxa, for those taxa that have been included in the supraglottic pneumotype of the lung microbiome created by microaspiration of oral bacteria (Segal et al., 2016). From observed taxonomic profiles and distribution of pathogen and oral taxa abundances, we defined pathogen dominance or oral taxa dominance as relative abundance of >50%, respectively. We compared log-transformed concentrations of host-response biomarkers and pathogen or oral taxa dominance with linear regression models, adjusted for culture results and history of COPD.

### Integrative Analysis and Modeling of Microbiome and Clinical Data

To move beyond simple correlations and comprehensively examine which microbiome variables are directly linked to clinical variables, we used Probabilistic Graphical Models (PGMs). PGMs can estimate and graphically represent the complex relationships of large numbers of variables that interact with each other, allowing for the discovery of direct links between variables based on their conditional dependencies. We used the *CausalMGM (Causal Mixed Graphical Model)* R package^1^, a novel algorithm that can accurately identify the underlying graphical model structure over mixed data types (continuous and discrete) (Sedgewick et al., 2016; Raghu et al., 2017).

## Results

### Cohort Descriptive Data

Fifty six patients were enrolled (mean age 56 years, 61% men), 12 (21%) with positive cultures for common respiratory pathogens (**Table 1**). Culture-positive and negative patients had similar distribution of comorbid conditions, severity of illness scores, mechanical ventilation parameters, laboratory values and clinical outcomes (**Table 1**). Empiric antibiotics were prescribed for 54/56 (96%) of patients at the time of enrollment, with a median exposure of two different classes of antibiotics (gram-positive, gram-negative, and atypical coverage).

**Table 1:** Baseline characteristics and clinical outcomes of patients enrolled in the Microbiome Cohort of the Acute Lung Injury Registry (MICALIR) study, categorized as patients with positive or negative respiratory cultures.

Variable	All	Culture-positive	Culture-negative^^^	*P*-value
N	56	12	44	
Age, mean (*SD*), years	55.9 (15.3)	54.7 (17.2)	56.2 (14.9)	0.88
Males, N (%)	34 (61)	5 (42)	29 (66)	0.18
BMI, mean (*SD*)	32.2 (10.2)	28.8 (7.1)	33.1 (10.8)	0.19
History of diabetes, N (%)	25 (45)	6 (50)	19 (43)	0.75
History of COPD, N (%)	17 (30)	5 (42)	12 (27)	0.47
History of pulmonary fibrosis, N (%)	4 (7)	1 (9)	3 (7)	1.00
History of smoking, N (%)				1.00
Current	15 (27)	3 (25)	12 (27)	
Former	15 (27)	3 (25)	12 (27)	
Never	26 (46)	6 (50)	20 (46)	
Sepsis, N (%)^#^	50 (89)	12 (100)	38 (86)	0.32
ARDS, N (%)^$^	21 (38)	7 (58)	14 (32)	0.11
High clinical index for pneumonia^&^	34 (61)	12	22 (50%)	**0.002**
Aspiration, N (%)	14 (25)	3 (25)	11 (25)	1
SOFA score, median (IQR)^∗^	7.0 (4.8–9.0)	8.5 (6.8–9.2)	7.0 (4.0–9.0)	0.09
PaO_2_:FIO_2_ ratio, mean (*SD*), mmHg	168.7 (81.6)	172.2 (118.3)	164.6 (67.2)	0.51
PEEP, median (IQR), cm	5.0 (5.0–10.0)	5.0 (5.0–9.0)	5.0 (5.0–10.0)	0.48
Plateau pressure, mean (*SD*), cm	23.3 (7.3)	23.2 (8.8)	23.6 (7.1)	0.90
Tidal volume (per kg of PBW), mean (*SD*), ml/kg	6.7 (1.2)	6.5 (1.5)	6.7 (1.1)	0.64
SBP, mean (*SD*), mmHg	118.7 (20.4)	105.2 (14.8)	122.3 (20.3)	**0.007**
Creatinine, median (IQR), mg/dl	1.4 (0.8–2.4)	1.9 (1.2–3.6)	1.4 (0.8–2.4)	0.26
WBC, mean (SD), x 10^-9^ per liter	13.9 (6.1)	15.8 (6.5)	13.4 (5.9)	0.33
Temperature, mean (*SD*), C	37.1 (0.9)	36.9 (0.9)	37.1 (0.9)	0.42
Respiratory Virus infection, N (%)	6 (11)	2 (17)^^^^	4 (10) ^##^	0.58
ICU LOS, median (IQR), days	8.0 (6.0–15.0)	7.5 (6.0–18.2)	8.5 (5.8–14.2)	0.77
VFD, median (IQR), days	21 (0–24)	10.5 (0–23.2)	20.5 (9.5–24.2)	0.29
30 Day mortality, N (%)	13 (23)	4 (33)	9 (20)	0.44
Acute kidney injury, N (%)	44 (79)	11 (92)	33 (75)	0.42

*Data are presented as mean (with standard deviations) or median (with interquartile range) for continuous variables (for normally and not normally distributed variables, respectively) and N (%) for categorical variables. Values of recorded variables were collected within 24 h of enrollment. P-values for comparisons between patients with positive respiratory cultures vs. negative respiratory cultures are shown, obtained from non-parametric Mann–Whitney test comparisons for continuous variables and Fisher tests for categorical variables. Statistically significant p-values (p < 0.05) are highlighted in bold. SD, standard deviation; IQR, interquartile range; BMI, body mass index; COPD, chronic obstructive pulmonary disease; ARDS, acute respiratory distress syndrome; SOFA, sequential organ failure assessment; PaO_2_, partial pressure of arterial oxygen; FiO_2_, Fractional inhaled concentration of oxygen; WBC, white blood cell count; PBW, predicted body weight; PEEP, positive end-expiratory pressure; SBP, systolic blood pressure; ICU LOS, intensive care unit length of stay; VFD, ventilator free days. ^^^ For 6 of the 44 culture-negative patients, there were no available respiratory sample clinical cultures within 48 h of research sample acquisition. These patients had negative routine clinical screening swabs for methicillin-resistant *S. aureus* and Vancomycin-resistant Enterococcus and were retrospectively deemed as low index of suspicion for pneumonia (e.g., intubated for airway protection for seizures or drug overdose). For these cases, we assigned the absence of a clinical respiratory microbiologic specimen as negative cultures. ^#^Sepsis was defined according to the Sepsis-3 criteria. ^$^ARDS was diagnosed according to the Berlin definition criteria. ^∗^SOFA score calculation does not include the neurologic component of SOFA score because all patients were intubated and receiving sedative medications, impairing our ability to perform assessment of the Glasgow Coma Scale in a consistent and reproducible fashion. ^^^^^^ N = 2, respiratory syncytial virus and influenza. ^##^N = 4, influenza, respiratory syncytial virus, metapneumovirus and parainfluenza.*

### 16S Sequencing Results

**FIGURE 1:**
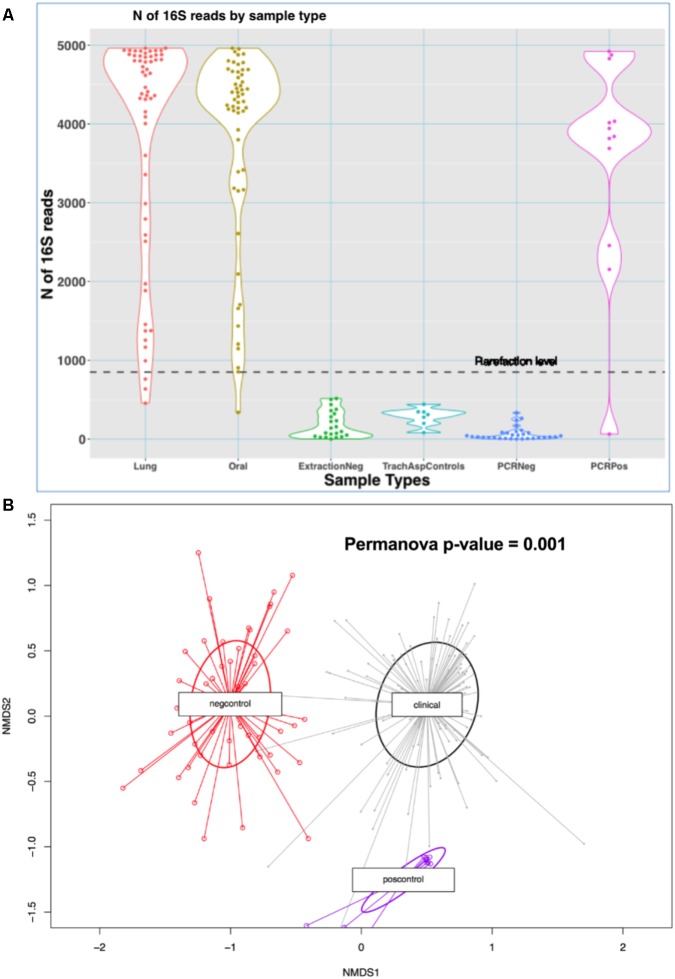
Comparisons of sequencing results between clinical samples and experimental controls. **(A)** N of reads by sample type in the MICALIR study. Clinical samples (lung and oral) produced about 30 times more reads than negative control samples (*p* < 10^-16^) [ExtractionNeg: negative control for DNA extraction experiments; TrachAspControls: sterile left-over saline from the one used to instill into the endotracheal tube for suctioning endotracheal aspirates; PCRNeg: PCR negative controls]. A rarefaction level of 850 reads was selected for alpha diversity analyses, which excluded small numbers of clinical samples for these analyses. **(B)** Non-metric multidimensional scaling (NMDS) plot of Bray–Curtis dissimilarity indices between clinical samples (lung and oral) versus negative controls (extraction, PCR and tracheal sampling procedure controls) and positive controls. Experimental control samples were compositionally markedly dissimilar to clinical samples by Bray–Curtis indices (Permanova *p*-value = 0.001). No taxa detected in negative controls were filtered from downstream analyses.

We analyzed a total of 110 clinical microbiome samples (56 ETAs and 54 oral swabs) and 71 experimental control samples. Clinical samples produced 3835 (standard deviation [*SD*] = 1329) reads (high-quality 16S sequences) on average, whereas negative experimental controls generated relatively few reads (mean = 136 [*SD* = 146]) (*p* < 10^-16^) (**Figure 1A**) and were compositionally dissimilar to clinical samples by Bray–Curtis indices (**Figure 1B**).

### Lung Microbial Community Profiles

**FIGURE 2:**
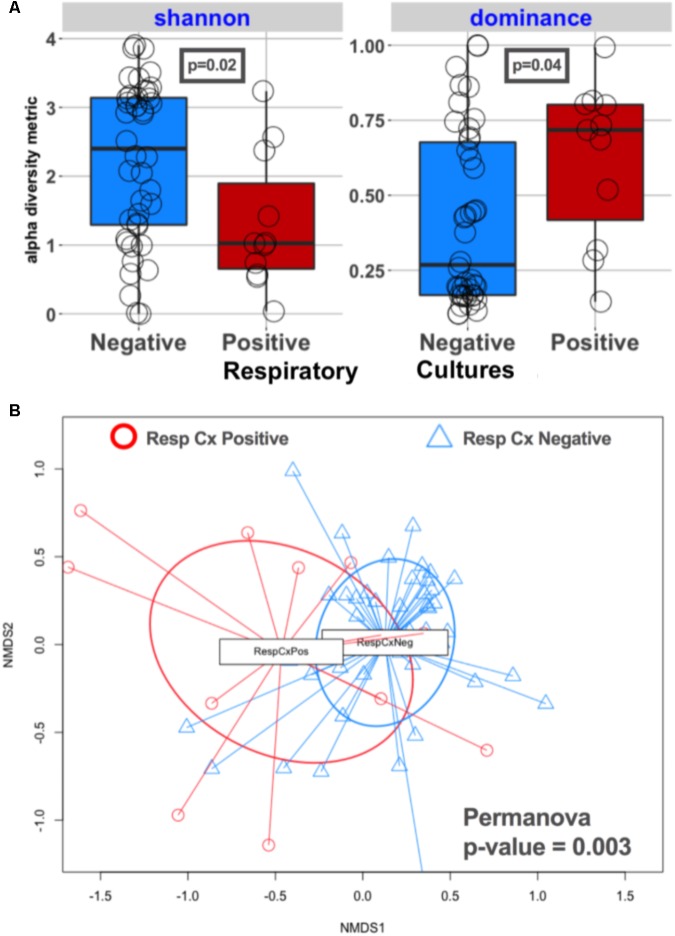
Culture-positive lung communities have significantly lower alpha diversity and are compositionally distinct from culture-negative communities. **(A)** Alpha diversity comparisons between culture-positive and culture-negative cases showed statistically significant differences in Shannon and Dominance indices, representing richness and evenness, respectively, indicating that culture-positive communities are being dominated by fewer bacterial species. Culture-negative communities demonstrated a wide range of alpha diversity. **(B)** Beta diversity (Bray–Curtis dissimilarity indices) comparisons between respiratory culture-positive (Resp Cx Positive) and negative (Resp Cx negative) cases showed significant differences (Permanova *p*-value = 0.003), but certain culture-negative samples overlapped with the culture-positive cluster indicating underlying compositional similarity.

Lung communities in culture-positive subjects had significantly lower alpha diversity (richness [*p* = 0.02] and evenness [*p* = 0.04]) compared to culture-negative subjects (**Figure 2A**). Culture-negative communities had a wide distribution of alpha diversity, ranging from very low richness as in culture-positive cases (Shannon = 0-1) to high alpha diversity (Shannon > 2.9) in the range of the healthy lung microbiome, despite the fact that these patients were exposed to broad-spectrum antibiotics (Morris et al., 2013). Culture-positive communities had overall significantly different taxonomic composition by Bray–Curtis indices compared to culture-negative communities (Permanova *p* = 0.003) (**Figure 2B**). On the other hand, lung communities from patients with a history of COPD (a disease process that is known to affect lung microbiome composition) (Sze et al., 2014) had modest differences in alpha and beta diversity compared to patients without COPD (Supplementary Figure 3).

**FIGURE 3:**
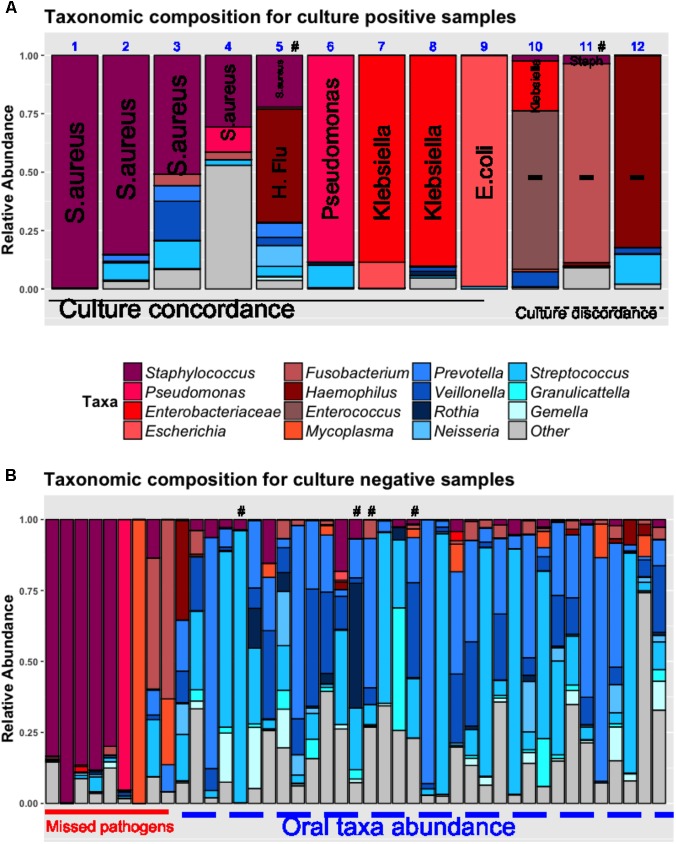
Pathogen dominance detection in culture-positive **(A)** and negative lung samples **(B)**. Taxonomic composition is shown as stacked bar-graphs, with each bar representing a patient’s community, with taxa colored individually and heights of component bars corresponding to relative abundance of each taxon. In culture-positive samples **(A)**, the clinically isolated organisms by routine microbiologic cultures are spelled out vertically in each bar (Methicillin-resistant *S. aureus* in cases 1, 2, 11; Methicillin-sensitive *S. aureus* in cases 3, 4, 5; *Haemophilus influenza* in case 5; *Pseudomonas aeruginosa* in case 6, *Klebsiella pneumoniae* in cases 7, 8, and 10, *Escherichia coli* in case 9; *Serratia marcescens* in case 12. In cases 1–9, the most abundant taxon corresponded to the clinically isolated pathogen (culture-concordance). In three cases (10–12), there was discordance between cultures and sequencing (i.e., the most abundant organism was not the one isolated by cultures. In cases 10 and 11, the clinically isolated *Klebsiella pneumoniae* and *S. aureus* corresponded to a minority of concordant reads in these communities that were dominated by *Enterococcus* and *Fusobacterium* taxa, respectively. In case 12, sequencing showed dominance by *Haemophilus* taxa whereas cultures isolated *Serratia marcescens*. Among culture-negative samples **(B)**, 20% were dominated by pathogenic taxa similar to the ones detected in culture-positive cases, and the remaining samples showed high abundance of oral bacteria. In six cases highlighted with the “#” symbol, respiratory viral panels of the nasopharynx or respiratory specimens were positive (for influenza, respiratory syncytial virus, metapneumovirus or parainfluenza virus). The “other” taxonomic assignment corresponds to multiple genera not corresponding to “pathogens” or “oral taxa” lumped together for display purposes. H.Flu, *Haemophilus Influenza*.

By examining the taxonomic composition of the 12 culture-positive samples (**Figure 3A**), we found that 16S sequencing detected taxa concordant to the clinically isolated bacterial pathogens in 11/12 samples (e.g., *Staphylococcus* genera for *S. aureus* and *Enterobacteriaceae* for *Klebsiella pneumoniae*). In 9 (75%) samples, the concordant taxa were the most abundant organisms in their respective communities. In two other cases (cases 10 and 11, **Figure 3A**), sequencing revealed that taxa corresponding to clinical isolates (*Klebsiella* and *Staphylococcus*, respectively) had low abundance in their communities, which were dominated by potentially pathogenic taxa undetected by clinical cultures (*Enterococcus* and *Fusobacterium*, respectively) (Grupper et al., 2009; Johannesen et al., 2016; Kelly et al., 2016). Although definitive causal inferences about the clinical impact of these bacteria are not possible, in the case of *Fusobacterium* dominance (case 11), vancomycin monotherapy targeted against the cultured methicillin-resistant *S. aureus* (MRSA) failed to produce a clinical response, whereas improvement ensued after empiric addition of piperacillin/tazobactam which in fact is effective against the fastidious anaerobe *Fusobacterium* (Johannesen et al., 2016).

Based on the overall concordance between culture-isolated bacteria and taxonomic abundance by sequencing, we operationally defined the most abundant taxa in culture-positive communities as “pathogens” (Supplementary Table 2 and Supplementary Figure 4). Looking then into the culture-negative cases that represent a diagnostic “black-box,” we found that 9/44 (20%) communities were dominated by pathogenic taxa (e.g., *Staphylococcus* or *Pseudomonas* genera) suggesting a specific etiology that was undefined by standard culture-based methods (**Figure 3B**). The remaining culture-negative samples were populated by the most common members of the supraglottic pneumotype of the lung microbiome, such as *Prevotella*, *Veillonella*, and *Streptococcus* taxa (“oral-origin” taxa) (Segal et al., 2016; Dickson et al., 2017). In four culture-negative cases, clinical viral panel testing was positive for respiratory viruses (influenza, parainfluenza, metapneumovirus, and respiratory syncytial virus) without identification of common bacterial pathogens (**Figure 2B**), and thus no indication of the commonly suspected bacterial super-infection that leads to empiric antibiotic courses in patients with viral pneumonia (van der Sluijs et al., 2010).

**FIGURE 4:**
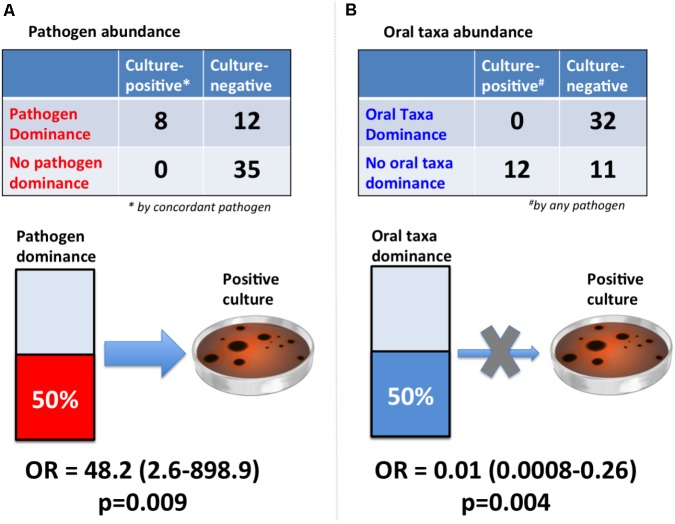
Dominance of lung communities by pathogens or oral taxa was strongly associated with respiratory culture results. **(A)** Pathogen dominance (>50% abundance) was strongly associated with concordant pathogen culture-positivity [Fisher’s odds ratio (OR) with continuity correction and associated 95% confidence interval shown]. The reference standard here was chosen to be concordant pathogen positivity, as the sequencing diagnostic test would be clinically valid if able to detect the same organism as our current reference standard of cultures. **(B)** Oral taxa dominance (>50% abundance) practically eliminated the odds of culture-positivity by any pathogen (OR = 0.01). The reference standard here was defined as “any pathogen,” given that oral taxa are not generally considered as pathogens or speciated by routine microbiologic cultures, and this comparison aims to assess the negative predictive value of high oral abundance in a lung community for ruling out culture positivity by any pathogenic bacteria.

After examining different thresholds of pathogen abundance (Supplementary Figure 5), we found that a relative abundance of ∼50% (community dominance) was strongly associated with bacterial culture results with an odds-ratio (OR) of 48.2 (95% CI: 2.6-898.9, *p* = 0.009) (**Figure 4**). In contrast, oral taxa dominance was strongly “protective” against respiratory culture positivity (OR = 0.01 [95% CI: 0.0008–0.26, *p* = 0.004]).

**FIGURE 5:**
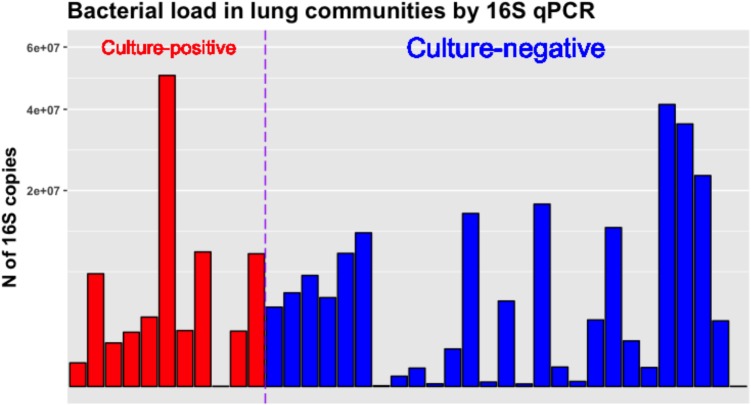
Bacterial load in culture-positive and negative lung communities as quantified by 16S rRNA gene qPCR. No significant differences between culture-positive and negative samples were found. The Y-axis showing number of 16S rRNA gene copies per sample is square-root transformed for visualization purposes.

We also quantified the bacterial load in lung communities by 16S qPCR and found no significant differences in absolute number of 16S rRNA gene copies between culture-positive and negative samples, underscoring the fact that several culture-negative samples were carrying high bacterial loads that were clinically undetected. There was a wide range of 16S rRNA copies across samples (range from 34 to 50,390,355 copies) (**Figure 5**), and we did not identify a diagnostic threshold of absolute pathogen abundance for association with culture positivity. Thus, in our dataset relative abundance of pathogens, which is reflective of within community microbial dynamics, appeared as a stronger predictor of culture positivity compared to absolute pathogen abundance as quantified by 16S qPCR copies.

### Oral Microbial Community Profiles

**FIGURE 6:**
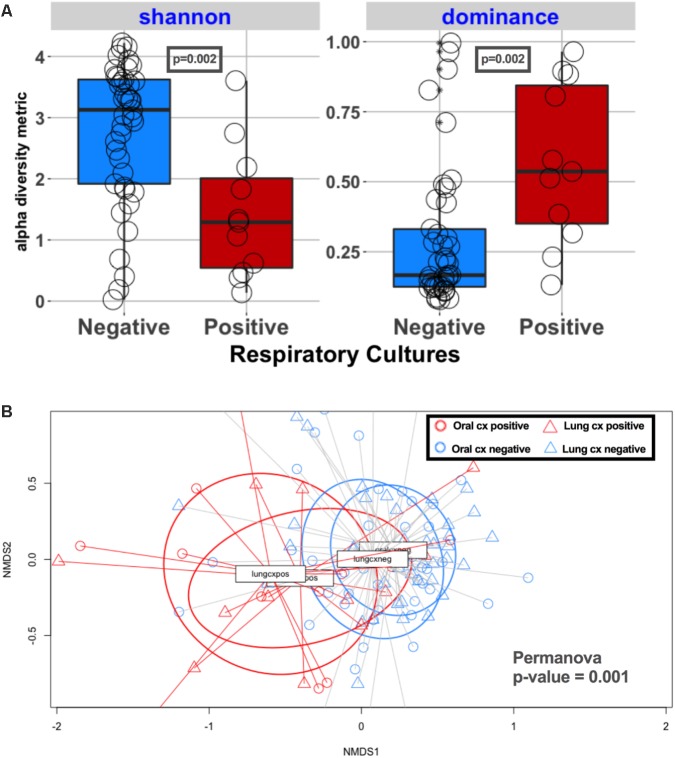
Alpha and beta diversity comparisons in oral and lung communities, stratified by respiratory specimen culture positivity. **(A)** Alpha diversity comparisons in oral communities, showing statistically significantly lower richness and evenness in culture-positive samples compared to culture-negative ones (*p* = 0.002). **(B)** Bray–Curtis dissimilarity indices comparison in 4 groups: red circles for oral communities of culture-positive samples, blue circles for oral communities of culture-negative samples, red triangles for lung communities of culture-positive samples, blue triangles for lung communities of culture-negative samples. Permanova indicates significant differences overall, but oral and lung communities are overlapping when stratified by respiratory sample culture positivity, indicating that oral communities in culture-positive cases were taxonomically more similar to their corresponding culture-positive lung communities, rather than the oral communities of culture-negative cases.

Oral microbiome profiles closely reflected the patterns observed with the lung microbiome, with lower alpha diversity in subjects with positive respiratory cultures compared to culture-negative subjects (*p* = 0.002) (**Figure 6**). We further examined the taxonomic composition of the oral communities in subjects with pathogen dominance in their lung communities (*n* = 19) and found that the oral communities were dominated by the same pathogen (Supplementary Figure 6) in seven subjects (36%), implicating colonization of the oral cavity as a potential source of pneumonia pathogens and suggesting the potential utility of oral sample sequencing for pneumonia diagnosis.

### Host Responses to Lung Microbiota and Clinical Outcomes

**FIGURE 7:**
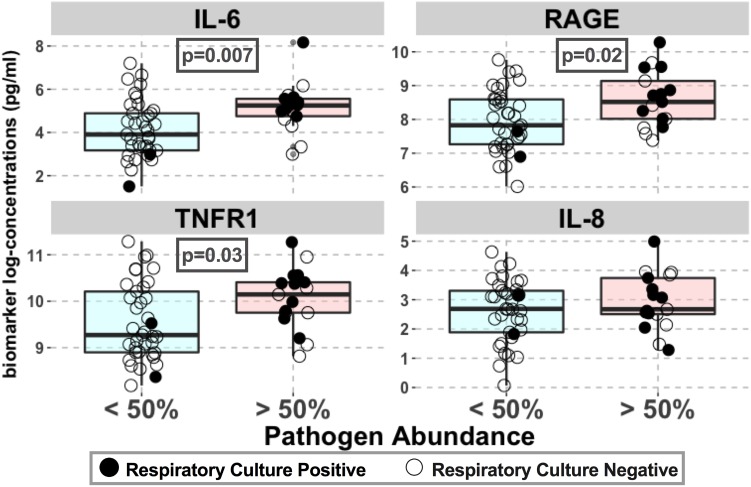
Pathogen abundance in lung communities was associated with higher levels of lung epithelial injury and inflammation. Associations between plasma biomarkers of injury (RAGE) and inflammation (IL-6, IL-8, and sTNFR1) with pathogen dominance in lung communities (relative abundance > 50%) are shown for the entire cohort. Biomarker concentrations (pg/ml) are shown in logarithmic scale. Culture-positive and negative samples are shown with filled and open circles, respectively. Statistically significant *p*-values are shown in boxes. No significant association with procalcitonin levels was found.

Lung community pathogen dominance was significantly associated with higher levels of circulating inflammatory cytokines (IL-6: *p* = 0.007; sTNFR1: *p* = 0.03) and epithelial injury biomarkers (RAGE: *p* = 0.02) (**Figure 7**). For IL-6, the association remained significant after adjusting for culture results (*p* = 0.03), suggesting that such pathogenic bacteria induce host inflammation regardless of their viability or ability to grow in cultures at the time of sample acquisition (**Table 2**). These associations remained statistically significant and with larger effect sizes when adjusted for history of COPD, a disease process that could also confound host-microbiome associations (**Table 2**). We did not find any significant associations between important patient-centered outcomes (mortality, shock, VFD and length of ICU stay) and pathogen dominance in the lung communities.

**Table 2:** Associations between pathogen abundance and host-response biomarkers in unadjusted linear regression models, and in adjusted models for culture positivity or history of COPD.

Biomarker	Coefficient and *p*-value		
	Unadjusted	Adjusted for culture positivity	Adjusted for history of COPD
**IL-6**	**1.02 (0.007)**	**0.99 (0.03)**	**1.07 (0.006)**
**sTNFR1**	**0.51 (0.03)**	0.47 (0.1)	**0.57 (0.02)**
**IL-8**	0.30 (0.32)	0.32 (0.39)	0.30 (0.34)
**RAGE**	**0.64 (0.02)**	0.56 (0.08)	**0.70 (0.009)**
**Procalcitonin**	0.56 (0.41)	0.75 (0.38)	0.85 (0.21)

*Statistically significant associations are highlighted in bold.*

### Network Analyses

**FIGURE 8:**
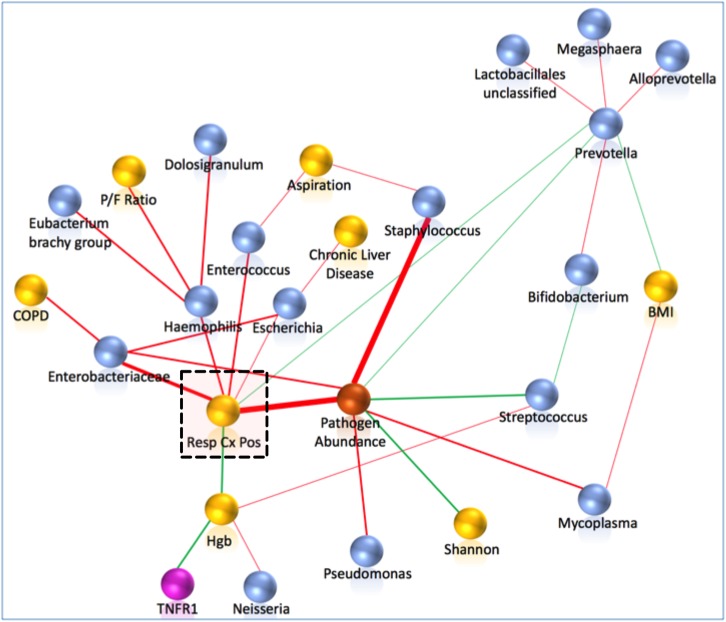
Pathogen abundance by sequencing was the strongest correlate of culture-positivity by probabilistic graphical modeling. Network analyses included clinical (yellow), individual 16S taxa (blue), composite pathogen taxa abundance (orange) and biomarker (purple spheres) variables. First and second neighbors around the clinically important variable of respiratory culture positivity (highlighted by a dashed square) are shown. Edges (links) between variables are shown in red for positive and in green for negative correlations. The thickness of the edges is proportional to the stability metric of the detected associations. Respiratory culture positivity was positively associated with the composite variable of pathogen abundance, Enterobacteriaceae, *Haemophilus*, *Escherichia*, and *Enterococcus* taxa, and negatively associated with *Prevotella* abundance and hemoglobin levels. COPD, chronic obstructive pulmonary disease; Hgb, hemoglobin; P/F Ratio, Pa02/Fi02 ratio; BMI, body mass index.

Probabilistic graph interrogation of our dataset using *CausalMGM* identified five microbial taxa and the levels of hemoglobin to be the most informative values for respiratory culture positivity, as well as a composite variable measuring pathogen taxa abundance (**Figure 8**). These findings extended our univariate taxonomic analyses in that they identified the taxa that are directly linked to positive cultures (not simply correlated) and highlighted sequenced bacterial taxa as the strongest explanatory variables of culture positivity. To examine the ability of 16S taxonomic data alone to predict culture positivity, we used the Markov blanket around the culture-positivity variable as a feature selection method (Huang et al., 2015). The taxonomy-based classifier yielded mean accuracy of 82.3% (*SD* = 7%) (**Table 3**), indicating proof-of-concept utility for use of sequencing in clinical practice for predicting culture results, if sequencing results were available real-time.

**Table 3:** Predictive taxa of culture positivity along with their numbers of appearance in the 10 Markov blankets.

Taxa	Number of Appearances
*Staphylococcus*	1
*Corynebacteriaceae_unclassified*	1
*Escherichia*	3
*Haemophilus*	3
*Enterococcus*	6
*Enterobacteriaceae*	8

*This predictive model with a linear weighted support vector machine classifier was able to correctly discriminate respiratory specimen culture positivity with taxonomic information only with a mean accuracy of 82.3% (SD = 7%).*

## Discussion

Our prospective study in mechanically ventilated patients provides the largest examination to date of the clinical validity of NGS for etiologic diagnosis of index severe pneumonia. By utilizing minimally invasive samples, we detected high abundance of pathogenic bacteria in both culture-positive and negative cases of pneumonia, which could allow for timely etiologic pathogen identification and antibiotic adjustments once technology evolves to allow rapid NGS. Our analyses highlighted overall community structure differences associated with culture-positivity and uncovered distinct differences in the host immune responses to dominant bacterial taxa in the lungs. Probabilistic graphical models that inclusively consider the wide range of variables in our dataset provided insights on the taxonomic and clinical variables directly linked to respiratory culture positivity. Our results indicate that respiratory microbiome profiles may provide clinically meaningful and actionable information above that currently afforded by standard microbiologic cultures.

16S sequencing provided important insights into the etiology of both culture-positive and negative bacterial pneumonias. In culture-positive cases, taxa concordant to clinically isolated pathogens dominated the respective communities in the vast majority of cases, consistent with the concept of community collapse during infection (Dickson et al., 2014). In the few exceptions of discordance between dominant pathogen and culture results (cases 10–12, **Figure 3A**), sequencing did find the cultured pathogen although it was not dominant and may have uncovered co-primary or alternative pathogens not clinically considered. Thus, even in cases of supposed diagnostic certainty, cultures may only capture a small portion of the underlying bacterial community and miss dominant bacteria that may have important therapeutic implications.

Culture-negative samples represent a diagnostic “black-box” in clinical practice, and in up to 75% of pneumonia cases no bacterial pathogen is ever isolated (Jain et al., 2015), similar to the culture-negative rate seen in our cohort. We found that approximately 20% of our culture-negative samples actually contained a predominance of pathogenic taxa similar to those observed in culture-positive specimens (e.g., *Staphylococcus* or *Pseudomonas*). In contrast, eighty percent of culture-negative samples without a dominant pathogen had high oral bacteria abundance, similar to what is often found in the lung microbiome and suggesting ongoing micro-aspiration of oral secretions around endotracheal tube cuffs in these patients (Kitsios and McVerry, 2018). In general, oral-origin taxa were associated with lower levels of host immune responses compared to pathogenic taxa. Nevertheless, oral taxa dominance cannot be uniformly considered as clinically innocuous, given significant interindividual variability in host responses and absolute bacterial load, making context-specific integration of clinical and microbiome data necessary for clarifying the clinical importance of individual oral bacteria.

Improvements in the etiologic diagnosis of pneumonia offered by NGS can translate into measurable benefits as defined by appropriate antibiotic targeting. For example, when a culprit pathogen is identified to dominate a community, early antibiotic tailoring against this pathogen becomes feasible and avoids the hazards of empiric broad-spectrum antibiotic courses. Furthermore, in up to 40% of culture-negative communities, no pathogenic sequence abundance was found and communities had high alpha diversity metrics in the range of the healthy human lung microbiome. With no indication of bacterial pneumonia in these patients by 16S sequencing, early discontinuation of antibiotics could substantially decrease their cumulative antibiotic exposure. Nonetheless, the 16S sequencing technique we utilized cannot yet be applied clinically and does not provide species-level or antibiotic resistance information. The advent of ultra-rapid sequencing devices and bioinformatics pipelines offer the capacity for whole metagenome sequencing in a matter of hours, theoretically allowing for bedside pathogen identification including viruses and fungi and antibiotic resistance prediction (Naccache et al., 2014; Judge et al., 2015; Schmidt et al., 2017). In a recent proof-of-concept study, sequencing with a portable, point-of-care device (Oxford Nanopore Technologies) was able to identify the culprit pathogens before a clinical microbiology lab, underlining that NGS may alleviate the time-consumption problem with traditional culture techniques (Pendleton et al., 2017). Whole metagenome sequencing techniques remain to be optimized for pathogen detection from biological samples containing substantial amounts of human DNA.

Apart from the technological challenges outlined above, clinical research in the field also has to overcome the formidable challenge of comparing a new sensitive test (NGS) against a standard-of-care (microbiologic cultures) that is not a gold-standard (Rutjes et al., 2007). Given that clinical cultures can effectively grow only a subset of cultivable bacteria (Venkataraman et al., 2015), conventional sensitivity/specificity analyses of NGS become obsolete. To overcome such diagnostic challenges, the clinical reference standard has to be refined, with synthesis of multi-level data (clinical, radiographic, biomarkers etc.) to be combined in a “construct gold-standard” for pneumonia diagnosis with supervised (involving expert input) or unsupervised classification methods (Rutjes et al., 2007). Our PGM analyses highlighted sequencing variables as the strongest predictors of culture-positivity. Through iterative training of machine-learning algorithms comparing sequencing profiles to “construct gold-standard” cases of pneumonia, a diagnostic, sequencing-based classifier can be developed for clinical use (Kitsios, 2018). Ultimately, demonstration of clinical efficacy of NGS-based testing for improving patient outcomes in randomized clinical trials will provide the evidentiary support necessary for clinical adoption.

The striking similarities between the oral and lung communities provided us with further understanding of the bacterial topography of the intubated respiratory tract (Kelly et al., 2016; Dickson et al., 2017; Kitsios et al., 2017). Although not part of routine clinical screening, oral cavity colonization by pathogenic gram-negative bacteria is a well-known harbinger of pneumonia in hospitalized patients (Dickson, 2016). Our analyses uncovered oral communities with dysbiosis (very low alpha diversity) in patients with positive sputum or BAL cultures. With cross-sectional examination of the respiratory microbiome, we could not assess for temporal bacterial immigration along the oro-tracheal tract. However, the observed patterns of oral and lung co-dominance by the same pathogens strongly indict the mouth as the originating pool of pathogens, which notably occurred despite routine oropharyngeal decontamination with chlorhexidine in our ICU (Price et al., 2014). Thus, sequencing of non-invasive oral swabs offers an attractive option for screening for pathogen colonization and for plausible pathogen detection in patients with pneumonia when lower respiratory specimens are unavailable.

The significant associations between sequencing-detected abundance of pathogens and host biomarkers of injury and inflammation (RAGE, IL-6, TNFR1) provided further validation of the biological and clinical relevance of sequencing profiles for pneumonia diagnosis. Consistent with other recent reports correlating lung microbiota composition with concurrently measured plasma biomarkers in critically ill patients (Panzer et al., 2017), our findings indicate that inter-individual heterogeneity in patients with severe pneumonia and ARDS may be explained on the basis of host-microbiome interactions, offering a new avenue for identifying clinical subphenotypes for personalized therapies (Calfee et al., 2014). Our linear regression models identified history of COPD as a significant confounder of host–microbiome associations, and thus COPD should be taken into account in future statistical modeling of these associations.

Our study has several limitations. It is a single-center design and is limited by available sample size, despite being the largest study of its kind. Consequently, the panel of pathogenic bacteria detected by sequencing is limited (Srinivasan et al., 2015), and generalizing to other critically ill populations and bacteria should be cautious (Calfee et al., 2014). *Streptococcus* taxa were commonly found in our patients with oral taxa-predominant bacterial communities, and without species-level information, we cannot exclude that certain 16S sequences belonged to pathogenic *Streptococcus pneumoniae* (albeit not recovered in any culture). However, in sensitivity analyses in which we coded *Streptococcus* sequences as “pathogens,” the associations between pathogen abundance and host responses were attenuated and no longer statistically significant (data not shown), suggesting that *Streptococcus* taxa detected in our samples are less likely to induce host inflammation and injury.

Since we performed 16S sequencing in extracted DNA, we did not consider viruses (either DNA or RNA) or fungi in our analyses, which can be important pathogens in subgroups of hospitalized patients (Jain et al., 2015). Sequencing of the bacteriome is nonetheless of paramount importance, even when a viral pathogen is identified, as the concern of bacterial super- or co-infection is pervasive among clinicians. For practical and ethical purposes, we did not perform BAL for microbiome analyses, thus we could not assess for regional variability of communities. Our approach to rely on ETAs was informed by the recent clinical practice guidelines by the American Thoracic Society and the Infectious Disease Society of America, recommending non-invasive testing via ETAs over invasive testing (with bronchoscopy or blind bronchial sampling) for hospital-acquired or ventilator-associated pneumonia diagnosis (Kalil et al., 2016). These recommendations are based on evidence from five randomized clinical trials (Berton et al., 2014), including a multicenter clinical trial (Canadian Critical Care Trials Group, 2006) showing that there is no clinical advantage (in terms of mortality, length of ICU stay, duration of mechanical ventilation or antibiotic management changes) between invasive or non-invasive sampling practices. For research purposes, ETAs are considered minimal-risk for enrolled patients, and represent a generally accepted “summary statement” of the pulmonary microbiome in intubated patients (Kelly et al., 2016; Kitsios et al., 2017; Panzer et al., 2017). Thus, we considered *a priori* that our comparisons between clinical BAL samples and research ETAs are internally valid for assessing concordance between cultures and sequencing. Our results showing striking concordance between dominance pathogen by sequencing with culture positivity (**Figure 4**) further validated the comparability of ETAs vs. BAL for microbiologic diagnosis. Timing differences of sample acquisition between clinical cultures and research ETAs may account for some of the observed sequencing-culture discordances, although research samples were obtained within 24hrs of clinical samples in >75% cases. Finally, we did not compare NGS against evolving techniques for rapid pathogen identification (Zumla et al., 2014), because cultures remain the current standard-of-care and allowed us to perform pragmatic comparisons in clinically challenging cases.

In summary, our study provides proof-of-concept evidence that as NGS technologies develop further, they will become useful as pneumonia diagnostic tools in the ICU to allow for fast and reliable etiologic pathogen identification. Our results demonstrate the clinical relevance of comprehensive microbial community profiling to provide information beyond what is currently available in clinical practice by microbiologic cultures and to directly impact clinical decision-making. Further prospective study in larger patient cohorts will allow for meaningful integration of sequencing output in culture-independent definitions of pneumonia.

## Data Availability Statement

All datasets generated and analyzed for this study are included in the Supplementary Files and are also publicly available for download at https://github.com/MicrobiomeALIR/Resp_Microbiome_Profiles_Pneumonia.

## Author Contributions

GK, AM, and BJM: conception and design. GK, AF, SR, DM, KL, SQ, JH, YZ, YD, JE, WB, JL, BM, PB, AM, and BJM: acquisition, analysis or interpretation of data. GK, JE, WB, JL, AM, and BJM: clinical cohort phenotyping. GK, AF, SR, DM, KL, SQ, JH, YZ, YD, JE, WB, JL, BM, PB, AM, and BJM: drafting of work and/or revising for important intellectual content. GK, AF, SR, DM, KL, SQ, JH, YZ, YD, JE, WB, JL, BM, PB, AM, and BJM: final approval of version to be published; agreement to be accountable for all aspects of the work in ensuring that questions related to the accuracy or integrity of any part of the work are appropriately investigated and resolved.

## Conflict of Interest Statement

The authors declare that the research was conducted in the absence of any commercial or financial relationships that could be construed as a potential conflict of interest.

## Footnotes

1 https://github.com/benoslab/causalMGM

## Supplementary Material

DATA SHEET S1 Metadata file.

DATA SHEET S2 Combined metadata and taxonomic table.

DATA SHEET S3 Taxa table at genus level.

DATA SHEET S4 Extended Methods and Results.
