# Supplementary material for: Respiratory Microbiome Profiling for Etiologic Diagnosis of Pneumonia in Mechanically Ventilated Patients
Source: Front Microbiol. 2018 Jul 10;9:1413. doi: 10.3389/fmicb.2018.01413 (PMC6048198; doi:10.3389/fmicb.2018.01413)
Supplement: DATA SHEET S4 — Extended Methods and Results. [file Data_Sheet_4.docx]

**Respiratory microbiome profiling for etiologic diagnosis of pneumonia in mechanically ventilated patients.**

**Supplementary Material**

**Contents:**

- STROBE statement: pages 2-5
- Extensive Methods: pages 6-12
- Results - Supplementary Tables and Figures: pages 13-18
- R code used for analyses: 19-28
- References: 29-31
- **Supplementary Table 1. STROBE Statement—Checklist of items that should be included in reports of *cohort studies***

|  | Item No. | Recommendation | Page  No. | Relevant text from manuscript |
| --- | --- | --- | --- | --- |
| **Title and abstract** | 1 | (*a*) Indicate the study’s design with a commonly used term in the title or the abstract | 2 | “we prospectively collected…” |
|  |  | (*b*) Provide in the abstract an informative and balanced summary of what was done and what was found | 2 | “Thus, enhanced detection of pathogenic bacteria by sequencing improves etiologic diagnosis of pneumonia, correlates with host responses, and offers substantial opportunity for individualized therapeutic targeting and antimicrobial stewardship.” |
| Introduction | | | |  |
| Background/rationale | 2 | Explain the scientific background and rationale for the investigation being reported | 3 | “…Despite the theoretical advantages of NGS, the technology has not yet been validated as a diagnostic tool to guide antimicrobial prescriptions in the ICU.” |
| Objectives | 3 | State specific objectives, including any prespecified hypotheses | 3-4 | “To examine the clinical validity (Khoury et al., 2003) of 16S sequencing for etiologic diagnosis of bacterial pneumonia in patients requiring mechanical ventilation, we conducted this proof-of-concept study with the Microbiome cohort in Acute Lung Injury Registry (MICALIR) at the University of Pittsburgh Medical Center (UPMC), assessing the upper and lower respiratory tract microbiome composition and its association with clinical diagnoses, host responses, and clinical outcomes.  “ |
| Methods | | | |  |
| Study design | 4 | Present key elements of study design early in the paper | 3 | “Study Design and Participants: We conducted a prospective cohort study” |
| Setting | 5 | Describe the setting, locations, and relevant dates, including periods of recruitment, exposure, follow-up, and data collection | 3 | “…from June 2015 - March 2017 enrolling consecutive adult patients in the Medical ICU with acute respiratory failure within 72 hours of intubation.” |
| Participants | 6 | (*a*) *Cohort study*—Give the eligibility criteria, and the sources and methods of selection of participants. Describe methods of follow-up | 3 | “Eligible patients were 18 years or older with acute respiratory failure requiring mechanical ventilation via endotracheal intubation. Exclusion criteria included inability to obtain informed consent, presence of tracheostomy, or mechanical ventilation for more than 72 hours..” |
|  |  |  |  |  |
| Variables | 7 | Clearly define all outcomes, exposures, predictors, potential confounders, and effect modifiers. Give diagnostic criteria, if applicable | 4 | “A consensus committee of clinical experts (GDK, JE, WB, JSL, AM, BJM) reviewed all available data for the enrolled patients and performed retrospective classifications of the etiology and severity their acute respiratory failure. Classifications were performed without knowledge of microbiome sequencing or host biomarker data and included sepsis, acute respiratory distress syndrome (ARDS), pneumonia or aspiration, and intubation for airway protection without risk factors for ARDS per established criteria.(Singer et al., 2016; Neto et al., 2016; ARDS Definition Task Force et al., 2012; Gong et al., 2005) Prospective clinical outcomes included 30-day mortality, duration of ICU stay, acute kidney injury,(Mehta et al., 2007) incident shock (defined as need for vasopressors) and ventilator-free days (VFD).(Kalanuria et al., 2014) |
| Data sources/ measurement | 8* | For each variable of interest, give sources of data and details of methods of assessment (measurement). Describe comparability of assessment methods if there is more than one group | *4* | *“*Laboratory analyses: We extracted bacterial DNA directly from oral swabs and ETAs and amplified the V4 hypervariable region of the 16S rRNA gene for sequencing on the Illumina MiSeq platform (Morris et al., 2013). We also performed qPCR of the V3-V4 region of the 16S rRNA gene to obtain absolute bacterial loads in each sample (Liu et al., 2012). For plasma biomarker analyses, we constructed a custom Luminex multi-analyte panel (R&D Systems, Minneapolis) (McKay et al., 2017) targeting biomarkers associated with pneumonia diagnosis (procalcitonin) and acute lung injury outcomes (RAGE: receptor of advanced glycation end-products, IL-6: interleukin-6, IL-8: interleukin-8, sTNFR1: soluble tumor necrosis factor receptor 1) (Calfee et al., 2014; Famous et al., 2017; Narvaez-Rivera et al., 2012). Clinical microbiologic cultures were processed per standard procedures as described in the Supplement.” |
| Bias | 9 | Describe any efforts to address potential sources of bias | Supplement | Positive and Negative control samples |
| Study size | 10 | Explain how the study size was arrived at | 5 | “56 patients were enrolled” |

| Quantitative variables | 11 | Explain how quantitative variables were handled in the analyses. If applicable, describe which groupings were chosen and why | 4-5 | “Data processing and statistical analysis methods: From derived 16S sequences, we applied a custom pipeline for Operational Taxonomic Units (OTUs-taxa) classification (Supplement)…pathogen or oral taxa dominance with linear regression models. |
| --- | --- | --- | --- | --- |
| Statistical methods | 12 | (*a*) Describe all statistical methods, including those used to control for confounding | 4-5 | As above |
|  |  | (*b*) Describe any methods used to examine subgroups and interactions | 4-5 | As above |
|  |  | (*c*) Explain how missing data were addressed | 4-5 | Complete case analysis. |
|  |  | (*d*) *Cohort study*—If applicable, explain how loss to follow-up was addressed |  | Not applicable |
|  |  | (*e*) Describe any sensitivity analyses | 9 | “However, in sensitivity analyses in which we coded *Streptococcus* sequences as “pathogens”, the associations between pathogen abundance and host responses were attenuated and no longer statistically significant (data not shown), suggesting that *Streptococcus* taxa detected in our samples are less likely to induce host inflammation and injury. |
| Results | | | | |
| Participants | 13* | (a) Report numbers of individuals at each stage of study—eg numbers potentially eligible, examined for eligibility, confirmed eligible, included in the study, completing follow-up, and analysed | 5 | Complete case analysis. |
|  |  | (b) Give reasons for non-participation at each stage |  | NA |
|  |  | (c) Consider use of a flow diagram |  | NA |
| Descriptive data | 14* | (a) Give characteristics of study participants (eg demographic, clinical, social) and information on exposures and potential confounders |  | Table 1. |
|  |  | (b) Indicate number of participants with missing data for each variable of interest |  | NA |
|  |  | (c) *Cohort study*—Summarise follow-up time (eg, average and total amount) |  | Table 1 – length of ICU stay |
| Outcome data | 15* | *Cohort study*—Report numbers of outcome events or summary measures over time |  | *Table 1.* |
| Main results | 16 | (*a*) Give unadjusted estimates and, if applicable, confounder-adjusted estimates and their precision (eg, 95% confidence interval). Make clear which confounders were adjusted for and why they were included |  | Figures 2, 4, 7 and Table 2 |
|  |  | (*b*) Report category boundaries when continuous variables were categorized |  | Supplementary Figure 5 |
|  |  | (*c*) If relevant, consider translating estimates of relative risk into absolute risk for a meaningful time period |  | NA |

| Other analyses | 17 | Report other analyses done—eg analyses of subgroups and interactions, and sensitivity analyses | 6-7 | | Host-response models and network analyses |
| --- | --- | --- | --- | --- | --- |
| Discussion | | | |  |  |
| Key results | 18 | Summarise key results with reference to study objectives | 7 | | “Our prospective study in mechanically-ventilated patients provides … the host immune responses to dominant bacterial taxa in the lungs. |
| Limitations | 19 | Discuss limitations of the study, taking into account sources of potential bias or imprecision. Discuss both direction and magnitude of any potential bias | 9 | | “Our study has several limitations. … to perform pragmatic comparisons in clinically challenging cases.” |
| Interpretation | 20 | Give a cautious overall interpretation of results considering objectives, limitations, multiplicity of analyses, results from similar studies, and other relevant evidence | 9 | | “In summary, our study provides proof-of-concept evidence that as NGS technologies develop further, they will become useful as pneumonia diagnostic tools in the ICU to allow for fast and reliable etiologic pathogen identification. Our results demonstrate the clinical relevance of comprehensive microbial community profiling to provide information beyond what is currently available in clinical practice by microbiologic cultures and to directly impact clinical decision-making. Further prospective study in larger patient cohorts will allow for meaningful integration of sequencing output in culture-independent definitions of pneumonia.” |
| Generalisability | 21 | Discuss the generalisability (external validity) of the study results | 9 | | Our study has several limitations. It is a single-center design and is limited by available sample size, despite being the largest study of its kind. Consequently, the panel of pathogenic bacteria detected by sequencing is limited,(Srinivasan et al., 2015) and generalizing to other critically ill populations and bacteria should be cautious.(Calfee et al., 2014) |
| Other information | |  | |  |  |
| Funding | 22 | Give the source of funding and the role of the funders for the present study and, if applicable, for the original study on which the present article is based | 10 | | **Funding**: National Institutes of Health (T32 HL007562 (GK); K23 HL139987 (GK); U01 HL098962 (AM); P01 HL114453 (BJM); R01 HL097376 (BJM); K24 HL123342 (AM); U01 HL137159 (DVM, PVB); R01 LM012087 (DVM, PVB); R01 HL086884 (JSL, WB); HL136143 (JSL, WB); F32 HL137258-01 (JE)) |

**Note:** An Explanation and Elaboration article discusses each checklist item and gives methodological background and published examples of transparent reporting. The STROBE checklist is best used in conjunction with this article (freely available on the Web sites of PLoS Medicine at http://www.plosmedicine.org/, Annals of Internal Medicine at http://www.annals.org/, and Epidemiology at http://www.epidem.com/). Information on the STROBE Initiative is available at http://www.strobe-statement.org.

**EXTENSIVE METHODS**

**Sample acquisition:**

Immediately after enrollment, we obtained baseline samples for microbiome analyses of the oral and lung communities and plasma samples for measurement of host-response biomarkers, with the following sample acquisition protocol:

**Oral swabs**: a sterile cotton swab was gently rubbed on the base of the tongue dorsum for ~ 5secs and then capped in the collection tube, labeled and frozen to -80°C as soon as possible until sample processing.

**Endotracheal aspirates (ETAs)**: Distal tracheal secretions were suctioned through a closed endotracheal tube suctioning system, similar to the ETAs obtained during routine clinical care for microbiologic culture studies. In cases that suctioning did not return adequate (>5ml) amount of ETAs, we instilled 5cc of sterile saline through the tubing system and then repeated suctioning. In cases where sterile saline was used for sampling, we also collected the left-over saline not used for sample collection (~ 5cc) as a negative control to examine for procedural contamination of our samples. Samples were directly collected in sputum collection traps, labeled and frozen to -80°C as soon as possible until sample processing.

**Plasma samples**: At the same time of respiratory sample collections, we also collected blood samples (10cc) (through central venous or arterial access or phlebotomy) collected in sodium citrate anticoagulated tubes (“blue-top”) provided that the patients’ hemoglobin concentration was >8g/dl to avoid risks of iatrogenic anemia. Samples were then transferred to the research laboratory for same day centrifugation and plasma collection and storage to -80°C.

**Clinical data recording:**

For each patient, we recorded detailed data abstracted from the electronic medical record (EMR) that were used in downstream analyses. Demographic and medical history data were collected from the EMR on the day of enrollment. Vital signs, laboratory results, mechanical ventilation parameters and chest radiography within 24hrs from enrollment were reviewed and the physiologically worse values (e.g. lowest blood pressure, or highest creatinine value) were recorded.

Demographic information: age, sex, height, weight, body mass index.

Pertinent medical history data: history of cardiac arrest, diabetes, chronic obstructive lung disease, congestive heart failure, chronic kidney disease, active neoplasm, chronic liver disease, history of pulmonary fibrosis, solid organ transplant, active immunosuppression.

Vital signs: temperature, heart rate, systolic blood pressure (lowest), mean arterial pressure (lowest).

Laboratory results: white blood cell count, hemoglobin, platelets, carbon dioxide serum concentration, glucose, arterial pHa, partial pressure of oxygen concentration, partial pressure of carbon dioxide concentration.

Mechanical ventilation parameters: respiratory rate, tidal volume per kilogram ideal body weight, positive end-expiratory pressure, peak inspiratory pressure.

Chest radiography: presence of bilateral infiltrates, abnormal chest x-ray at enrollment.

Clinical microbiology results: We considered clinical microbiologic results from samples obtained within 48hrs of research sample timing acquisition so that such samples would be reflective of the same infectious process being studied by next-generation sequencing. Clinical cultures were obtained at the discretion of the treating physicians who were not involved in the MICALIR study. We considered microbiologic cultures of respiratory specimens (sputum, ETA or bronchoalveolar lavage - BAL) as positive when pathogenic bacterial species had been isolated by the clinical laboratory. Cultures were considered as negative when no organism growth was observed or presence of “normal respiratory flora” was reported, as per standard clinical practices. For blood cultures, we considered results as positive when there was reported growth of organisms not deemed as skin contaminants by the treating physicians. We also recorded results of respiratory viral panel testing performed in nasopharyngeal swabs or sputum/BAL samples.

Severity of illness scores: sequential organ failure assessment (SOFA) score (calculation does not include the neurologic component of SOFA score because all patients were intubated and receiving sedative medications, impairing our ability to perform assessment of the Glasgow Coma Scale in a consistent and reproducible fashion). SOFA score was calculated by using the physiologically worse values within 24hrs of enrollment.

Medication administration: We recorded antibiotics and vasopressors administered during the first week of ICU course from intubation.

Outcomes: length of ICU stay, ventilator-free days (VFD), 30-day mortality, incident shock and occurrence of acute kidney injury (AKI) within the first week of ICU stay.

**Clinical Phenotyping:**

A consensus committee of clinical experts (GDK, JE, WB, JSL, AM, BJM) reviewed all available clinical, laboratory, microbiologic and radiographic data for the enrolled patients and performed retrospective classifications of their acute respiratory failure in terms of etiology and severity. All classifications were performed blinded to microbiome sequencing and host biomarker data, following implicit review of the available data and reaching consensus among the committee members. We diagnosed sepsis,(Singer et al., 2016) acute respiratory distress syndrome (ARDS),(ARDS Definition Task Force et al., 2012) pneumonia or aspiration,(Gong et al., 2005) and intubation for airway protection without risk factors for ARDS (Neto et al., 2016) per established criteria.

**Laboratory methods:**

Positive and Negative experimental control samples:

We considered three types of experimental negative control samples used to assess for possible contamination events from sample collection to DNA extraction and PCR amplification:

1. **Endotracheal aspirate controls**: In cases where sterile saline was used for sampling of ETAs, we collected the left-over saline not used for sample collection (~ 5cc) as a negative control to examine for contamination of the ETA from bacterial populations present in what is clinically considered sterile saline syringes. We extracted DNA from these saline samples and performed PCR amplification and 16S rRNA gene sequencing as performed for all clinical samples.
2. **DNA extraction negative controls**: We added DNA-free sterile water in one extraction column per each batch of clinical sample DNA extractions (~12-15 samples per experiment).
3. **PCR-negative controls**: We added DNA-free sterile water in the PCR reaction mix in amount equal to the amount of template DNA from clinical samples used (typically 6 microliters).

As PCR amplification positive controls, we utilized the ZymoBIOMICS Microbial Community DNA Standard (Zymo Research, Irvine, CA), a mock microbial community consisting of genomic DNA of eight bacterial strains. We utilized 1 microliter of the genomic mixture with concentration of 10ng/microliter for each reaction.

DNA extractions: We extracted bacterial DNA directly from samples (oral swabs and endotracheal aspirates - ETAs) using the Powersoil (MoBio) extraction kit following the manufacturer’s instructions, as previously described.(Morris et al., 2013) Due to high viscosity of ETA samples, we pretreated them with Dithiotreitol (0.1% DTT in phosphate buffered saline) in 1:1 dilution in order to dissolve the mucus and allow usability in DNA extraction columns.

16S rRNA gene sequencing: We amplified extracted DNA by PCR using the method of Caporaso et al.(Caporaso et al., 2012) and the Q5 HS High-Fidelity polymerase (NEB) targeting the V4 hypervariable region of the 16S rRNA gene. We utilized reagent controls for each step of the process (DNA extraction and PCR amplification). We amplified four microliters per reaction of each sample with a single barcode in triplicate 25 microliter reactions. Cycle conditions were 98˚C for 30s, then 33 cycles of 98˚C for 10s, 57˚C for 30s, 72˚C for 30s, with a final extension step of 72˚C for 2 min. We combined triplicates and purified with the AMPure XP beads (Beckman) at a 0.8:1 ratio (beads:DNA) to remove primer-dimers. We quantitated eluted DNA on a Qubit fluorimeter (Life Technologies). We performed sample pooling on ice by combining 20 ng of each purified band. For negative controls and poorly performing samples, we used 20 microliters of each sample. We purified the sample pool with the MinElute PCR purification kit. The final sample pool underwent two more purifications – AMPure XP beads to 0.8:1 to remove all traces of primer dimers and a final cleanup in Purelink PCR Purification Kit (Life Technologies). We quantitated the purified pool in triplicate on the Qubit fluorimeter prior to preparing for sequencing. We prepared the sequencing pool according to instructions by Illumina, with an added incubation at 95˚C for 2 minutes immediately following the initial dilution to 20 picomolar. We then diluted the sequencing pool to a final concentration of 7 pM + 15% PhiX control. Amplicons were sequenced on the Miseq platform.

16S rRNA gene quantitative PCR (qPCR): We performed qPCR reactions of the V3-V4 region of the 16S rRNA gene to quantify the bacterial load in each sample, with the BactQuant protocol (Liu et al., 2012). PCR reactions were prepared in a total volume of 20µL, which consisted of 2µL of 10XPCR buffer, 3.5 mmol/L MgCl_2_, 0.2 mmol/L deoxynucleoside triphosphate, 0.5 µmol/L forward and reverse primers, 0.225 µmol/L probe, 0.75 U of Platinum Taq polymerase (Invitrogen), and 2µL of each DNA.  The forward and reverse primers and the probe sequences that were used to amplify DNA templates encoding the V3-V4 region of the 16S rRNA gene were identical to those previously described (Liu et al., 2012).  The DNA was amplified in duplicate, and mean values were calculated. A standard curve was created from serial dilutions of plasmid DNA containing known copy numbers of the template.  The assays were performed on the LightCyler System (Roche) using the following PCR conditions: 95°C for 5 min, followed by 50 cycles at 95°C for 15 s and at 60°C for 1 min.

Reproducibility of sequencing results:

We demonstrated excellent reproducibility of taxonomic results with our 16S rRNA gene sequencing protocol in our lab. In periodic quality control experiments, we run technical replicates by using three different sets of primers (each one with different barcodes) to assess reproducibility of sequencing. In a recent set of 80 stool samples run in triplicate, we performed permutation analysis of variance (Permanova at 1000 permutations) by including the “technical replicate” variable as an explanatory variable for the variance in beta-diversity of samples. The technical replicate value explained a very small proportion of between sample variance (R2=0.0014) with a p-value of 0.99, indicating that no significant differences between replicated samples could be found. This was further illustrated with visualization by nonmetric-multidimensional scaling with demonstration of all samples and their centroids by technical replicate factor (Replicate 1 vs. 2 vs. 3) showing near-perfect overlap of the centroids, again suggesting of near-perfect similarity of the technical replicate samples in terms of beta-diversity.

**Supplementary Figure 1: Nonmetric-multidimensional scaling of technical replicate samples (each stool sample run in triplicate: R1 vs. R2 vs. R3) showing near perfect overlap of the centroids of these replicate samples, indicating excellent reproducibility of technical replicate samples.**


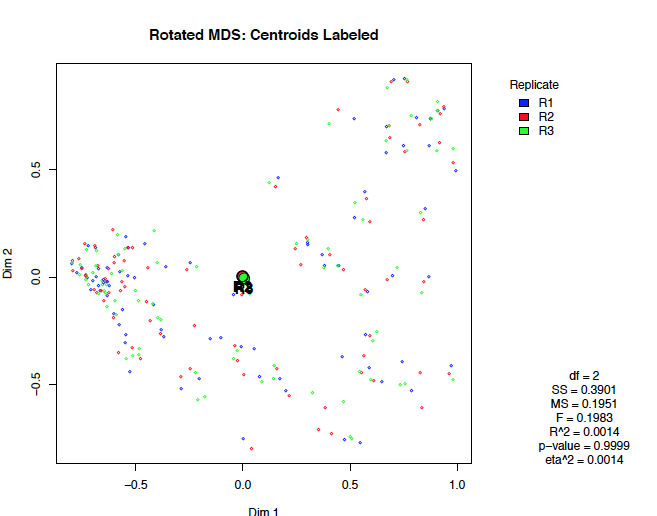


Additionally, our method accurately reproduced the taxonomic composition of Zymo samples, detecting the expected relative abundances of the eight component organisms in the ZymoBIOMICS Microbial Community DNA Standard (Zymo Research, Irvine, CA) (Supplementary Figure 2).

**Supplementary Figure 2: Taxonomic composition of 5 ZymoBIOMICS Microbial Community DNA Standard samples, which showed the expected relative abundances of the eight component taxa.**

**
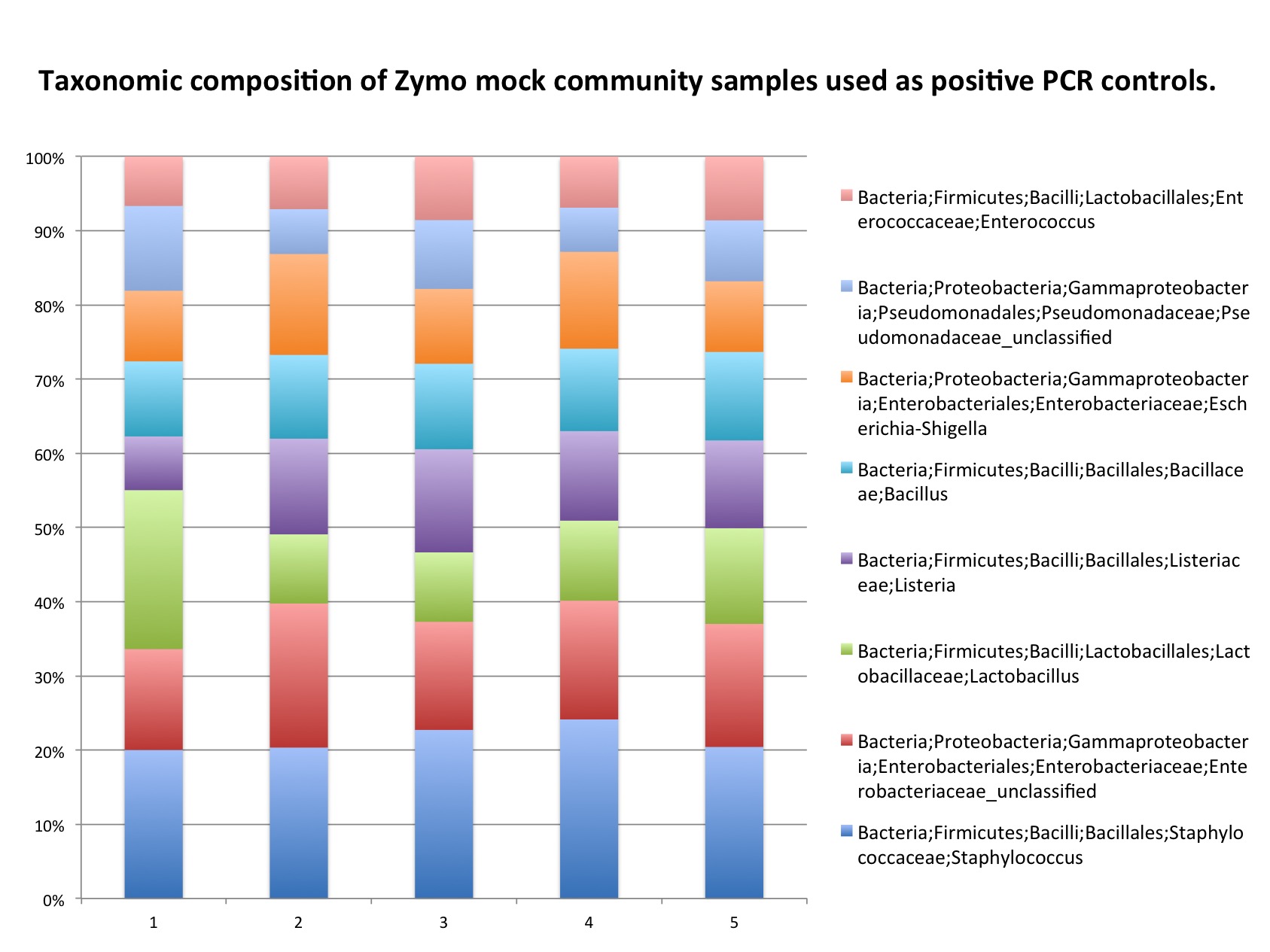
**

Microbial culture protocols:

All respiratory specimen cultures were performed by the clinical microbiology laboratory at the University of Pittsburgh Medical Center. Upon receipt of clinical specimens in the laboratory, the available material (sputum, endotracheal aspirate or bronchoalveolar lavage fluid) is used to inoculate agar plates: trypticase soy agar with 5% sheep blood, chocolate agar (for fastidious organisms), Columbia Naladixic Acid Agar (CAN Agar for gram-positives) and MacConkey (for gram-negatives). Samples are incubated for up to 24hrs and the following day, isolated colonies are identified with matrix-assisted laser desorption/ionization with time-of-flight mass spectroscopy (MALDI-TOF MS) (Bruker Biotyper) and susceptibility testing is performed with the MicroScan WalkAway platform (Beckman Coulter).

**Analytics**:

16S Sequence quality control:

Sequences from the pooled sequencing run were demultiplexed into individual sample/replicate fastq files. Each fastq file was then processed through the Center for Medicine and the Microbiome (CMM) custom modular read QC pipeline that was configured to perform the following steps: low complexity filtering, QV trimming, Illumina sequencing adapter trimming, and 16S primer trimming. Low complexity filtering utilized NCBI BLAST's dustmasker (Morgulis et al., 2006). Reads with greater than 80% low complexity regions were filtered. QV trimming and filtering utilized the FASTX Toolkit. The trimming threshold was QV>25 from the 3' end, and length>125 bp after trimming. The subsequent filtering threshold was >25 across >95% of the read. The Illumina sequencing adapter and 16S primer trimming was performed with cutadapt (Haas et al., 2011).

16S Clustering and annotation**:**

Paired sequences with forward and reverse reads passing the QC filtering and trimming steps were then mated (end aligned and a consensus sequence computed) using the make.contigs function of Mothur. Consensus sequences were screened to limit the overlap mismatch to no more than 20%. The maximum number of N's allowed in the overlap was 4 and the minimum overlap was required to be greater than 25bp. Consensus sequences passing screening were then passed through CMM's 16S clustering and annotation pipeline, a Mothur-dependent wrapper designed to streamline and automate the execution of the following Mothur steps: unique.seqs, align.seqs, screen.seqs, filter.seqs, second uniq.seqs, pre.cluster, chimera.uchime, remove.seqs, classify.seqs, dist.seqs, cluster, make.shared, and classify.otu. Mothur output files were then reformatted to sample x category (taxonomic levels or OTU) matrices for downstream statistical modeling (Schloss et al., 2009).

Taxa table edits:

The taxa table was filtered for low abundance taxa (relative abundance, <0.005%) and singletons, and also for samples that generated fewer than 650 reads in the range of negative control samples. One lung sample was filtered with <650 reads, and inspection of the highly diverse community indicated presence of contamination due to low biomass samples. We did not filter clinical samples for any taxa detected in the negative control samples.

Integrative analysis and modeling of microbiome and clinical data

To comprehensively examine potential relationships between clinical and microbiome variables not considered by our hypothesis-driven testing methods, we used Probabilistic Graphical Models (PGMs). PGMs offer a robust and attractive solution to estimate the joint probability distribution and represent in a comprehensive way the conditional (in)dependencies of the dataset’s variables. Knowing the graphical model structure one can extract useful information to help in disease prognosis, diagnosis, patient stratification, etc. By definition, in a directed graph, a variable *A* is independent of any other variable (or combinations of variables) in the graph conditioning on its Markov blanket (i.e. the parents, children and spouses of variable *A*). This is why Markov blankets have been successfully used in the past for feature selection(Aliferis et al., 2010a, 2010b) and why these directed graphs are referred to as “causal” in the literature. To comprehensively analyze our dataset, we used *CausalMGM (Causal Mixed Graphical Model)* <https://github.com/benoslab/causalMGM)>*,* a novel algorithm which can identify with accuracy the underlying graphical model structure over mixed data types (continuous and discrete).(Sedgewick et al., 2016) We used the R package *Causal-MGM* for this analysis.

To investigate the predictive ability of sequencing-derived information from the graphical models to correctly discriminate respiratory sample culture positivity, we applied a k-fold cross-validation method (in our case k=10).(Hastie et al., 2009) K-fold cross-validation is a well-established technique that helps evaluate predictive models in cases where the amount of data is limited. In cross-validation we partition the original dataset into a *training* set, which is used to estimate the parameters of the model, and to a *test* set which is used to evaluate the prediction accuracy of the model.(Sedgewick et al., 2016) In our case (10-fold cross-validation) we created 10 different partitions of our original dataset. Each training set consisted of 48 patients (8 culture-positive and 40 culture-negative) and each test set consisted of 8 patients (4 culture-positive and 4 culture-negative). For each one of the 10 training datasets, we run *CausalMGM* (Causal Mixed Graphical Model) and we estimate the corresponding graph structure. Next, using the estimated graph structure we identified the features that belong to the Markov blanket (Pattern Recognition and Machine Learning | Christopher Bishop | Springer) (i.e. parents, children and spouses) of the culture positivity variable and using their information we trained a linear weighted Support Vector Machine (SVM) classifier.(Hastie et al., 2009) It is worth to mention that the reason for training a *weighted* linear SVM classifier (instead of a linear SVM classifier) was to deal with the unbalance of the data in the corresponding training sets (only 8 culture-positive and 40 culture-negative patients). Following these steps, we evaluated the accuracy of the estimated classifier using the corresponding test set.

**RESULTS**

**Supplementary Figure 3: Alpha and beta diversity comparisons in lung communities stratified by history of COPD or smoking status.** Panel A. Alpha diversity comparisons by COPD history, showing that patients with history of COPD had lower richness (Shannon index) compared to patients without history of COPD (p=0.04). Panel B: Bray Curtis dissimilarity indices comparison between patients with COPD (purple circles) and patients without COPD (green triangles) showing modest differences (Permanova p-value = 0.02). Panel C: Alpha diversity comparisons by smoking status, showing no significant differences between current, former and never smokers. Panel D: Bray Curtis dissimilarity indices comparison by smoking status, showing no significant differences between current, former and never smokers.

**Supplementary Table 2. Definitions of pathogenic and oral origin taxa in the lung communities**. Taxa dominating culture-positive samples were considered as pathogens. *Mycoplasma* was also noted to dominate certain culture-negative samples and was also considered as a pathogen given that *Mycoplasma* isolation is considered pathogenic in clinical practice. Oral taxa were defined as the members of the supraglottic pneumotype of the healthy lung microbiome.(Segal et al., 2016) Genera belonging to the same taxon were grouped together. Pathogenic and oral taxa abundance was also expressed as the sum of relative abundances of the component pathogenic and oral taxa, respectively.

| **Pathogenic Taxa** | | **Oral Taxa** | |
| --- | --- | --- | --- |
| Group Taxon | Individual taxonomic assignments | Group Taxon | Individual taxonomic assignments |
| ***Staphylococcus*** | 1.Bacteria;Firmicutes;Bacilli;Bacillales;Staphylococcaceae;Staphylococcus  2.Bacteria;Firmicutes;Bacilli;Bacillales;Staphylococcaceae;Staphylococcaceae_unclassified | ***Prevotella*** | 1.Bacteria;Bacteroidetes;Bacteroidia;Bacteroidales;Prevotellaceae;Prevotella 2.Bacteria;Bacteroidetes;Bacteroidia;Bacteroidales;Prevotellaceae;Prevotella_2  3.Bacteria;Bacteroidetes;Bacteroidia;Bacteroidales;Prevotellaceae;Prevotella_6  4.Bacteria;Bacteroidetes;Bacteroidia;Bacteroidales;Prevotellaceae;Prevotella_7  5.Bacteria;Bacteroidetes;Bacteroidia;Bacteroidales;Prevotellaceae;Prevotella_9  7.Bacteria;Bacteroidetes;Bacteroidia;Bacteroidales;Prevotellaceae;Prevotellaceae_unclassified |
| ***Pseudomonas*** | 1.Bacteria;Proteobacteria;Gammaproteobacteria;Pseudomonadales;Pseudomonadaceae;Pseudomonas  2.Bacteria;Proteobacteria;Gammaproteobacteria;Pseudomonadales;Pseudomonadaceae;Pseudomonadaceae_unclassified | ***Veillonella*** | 1.Bacteria;Firmicutes;Negativicutes;Selenomonadales;Veillonellaceae;Veillonella  2.Bacteria;Firmicutes;Negativicutes;Selenomonadales;Veillonellaceae;Veillonellaceae_unclassified |
| ***Enterobacteriaceae*** | Bacteria;Proteobacteria;Gammaproteobacteria;Enterobacteriales;Enterobacteriaceae;Enterobacteriaceae_unclassified | ***Gemella*** | Bacteria;Firmicutes;Bacilli;Bacillales;Family_XI;Gemella |
| ***Escherichia*** | Bacteria;Proteobacteria;Gammaproteobacteria;Enterobacteriales;Enterobacteriaceae;Escherichia-Shigella | ***Granulicatella*** | Bacteria;Firmicutes;Bacilli;Lactobacillales;Carnobacteriaceae;Granulicatella |
| ***Fusobacterium*** | Bacteria;Fusobacteria;Fusobacteriia;Fusobacteriales;Fusobacteriaceae;Fusobacterium | ***Rothia*** | Bacteria;Actinobacteria;Actinobacteria;Micrococcales;Micrococcaceae;Rothia |
| ***Haemophilus*** | Bacteria;Proteobacteria;Gammaproteobacteria;Pasteurellales;Pasteurellaceae;Haemophilus | ***Neisseria*** | Bacteria;Proteobacteria;Betaproteobacteria;Neisseriales;Neisseriaceae;Neisseria |
| ***Enterococcus*** | Bacteria;Firmicutes;Bacilli;Lactobacillales;Enterococcaceae;Enterococcus | ***Streptococcus*** | Bacteria;Firmicutes;Bacilli;Lactobacillales;Streptococcaceae;Streptococcus |
| ***Mycoplasma*** | Bacteria;Tenericutes;Mollicutes;Mycoplasmatales;Mycoplasmataceae;Mycoplasma |  |  |

**Supplementary Figure 4. Clustering of lung microbiome samples based on Euclidean distances of taxonomic composition**. Six main clusters were identified: *Staphylococcus*, *Streptococcus*, *Pseudomonas*, Enterobacteriaceae, *Escherichia* and *other*. This clustering analysis (independent from the literature-based distinction into pathogens vs oral taxa) identified 4 major clusters of pathogenic taxa (*Staphylococcus*, *Pseudomonas*, Enterobacteriaceae and *Escherichia*) vs. *Steptococcus* and “other” bacteria, which mainly consisted of oral bacteria.

**Supplementary** **Figure 5. Distribution of relative abundance for pathogen (A) and oral taxa (B) in respiratory specimen culture positive and negative samples**. Culture-positive samples demonstrated a threshold of >50% relative pathogen abundance (A) and all had <50% oral taxa abundance (B). Based on these distributions, a threshold of 50% relative pathogen abundance was chosen for assessment of the diagnostic accuracy of sequencing for culture-positivity.

**Supplementary Figure 6. Taxonomic composition of (matched by patient) oral and lung communities in cases of pathogen dominance in the lung communities**. In 7/19 samples (36%) the oral community was also dominated by the same pathogenic taxa as the ones observed in the lung community (highlighted by asterisk). In one case, we were unable to obtain an oral swab (indicated with the minus sign).

**R Code used for analyses:**

| ---  title: "RespMicroProfiles_PNADX_FIM2018"  author: "Georgios Kitsios"  date: "6/22/2018"  output: html_document  ---  ###### R code for statistical and ecological analyses for the manuscript:  # "Respiratory microbiome profiling for etiologic diagnosis of pneumonia in mechanically ventilated patients."  # Author: Georgios D. Kitsios, MD, PhD  # load important libraries  library(dplyr)  library(readxl)  library(plyr)  library(ggplot2)  library(psych)  library(xlsx)  library(lubridate)  library(stargazer)  library(reshape2)  library(ggbeeswarm)  library(epitools)  library(vegan)  library(tidyr)  library(gdata)  ############ Metadata dataframe of baseline data for all 56 subjects included: "pnadx_meta"  # filter dataframe for lung samples only to allow for descriptive stats calculations  pnadx_meta_lung <- filter(pnadx_meta, sampletype=="tracheal")  # Obtain descriptive statistics for respiratory culture positive (balposbasl==1) and negative (balposbasl==0) patients.  # Use the library stargazer to automate calculations and output  stargazer(pnadx_meta_lung, type = "html", median = TRUE, iqr = TRUE, out = "...~USERDEFINED...")  # create a table that has all variables and then stratify tables by balposbasl vs not  # all - continuous variables  stargazer(subset(pnadx_meta_lung[c("Age", "BMI", "ICUstayduration", "VFD", "SOFAScore", "lips_score", "HR", "SBP", "Temperature", "LowestMAP", "PHa", "WBC", "Platelets", "Creatinine", "CO2", "WorstPFRatio", "TotalRR", "PEEP", "PeakInspiratory", "PlateauPressure", "TVmlperkg")]), type = "text", nobs=TRUE, mean.sd = TRUE, min.max = FALSE, median = TRUE, iqr = TRUE, title = "Descriptive statistics all", digits = 1, out = "...~USERDEFINED..."")  # all - categorical variable  stargazer(subset(pnadx_meta_lung[c("Gender", "HistDiabetes", "HistCOPD", "HistImmunosuppression", "HistCCF", "HistCRF", "IsActiveNeoplasm", "lips_pneumonia", "lips_sepsis", "lips_shock", "lips_aspiration", "Mortality30Day", "Mortality90Day", "AKIFirstWeek")]), type = "text", nobs=TRUE, mean.sd = TRUE, min.max = FALSE, title = "Descriptive statistics all categorical", digits = 2, out = "...~USERDEFINED..."")  # balposbasl - continuous variables  stargazer(subset(pnadx_meta_lung[c("Age", "BMI", "ICUstayduration", "VFD", "SOFAScore", "lips_score", "HR", "SBP", "Temperature", "LowestMAP", "PHa", "WBC", "Platelets", "Creatinine", "CO2", "WorstPFRatio", "TotalRR", "PEEP", "PeakInspiratory", "PlateauPressure", "TVmlperkg")], pnadx_meta_lung$balposbasl=="1"), type = "text", nobs=TRUE, mean.sd = TRUE, min.max = FALSE, median = TRUE, iqr = TRUE, title = "Descriptive statistics balpos", digits = 1, out = "...~USERDEFINED..."")  # balneg - continuous  stargazer(subset(pnadx_meta_lung[c("Age", "BMI", "ICUstayduration", "VFD", "SOFAScore", "lips_score", "HR", "SBP", "Temperature", "LowestMAP", "PHa", "WBC", "Platelets", "Creatinine", "CO2", "WorstPFRatio", "TotalRR", "PEEP", "PeakInspiratory", "PlateauPressure", "TVmlperkg")], pnadx_meta_lung$balposbasl=="0"), type = "text", nobs=TRUE, mean.sd = TRUE, min.max = FALSE, median = TRUE, iqr = TRUE, title = "Descriptive statistics balpos", digits = 1, out = "...~USERDEFINED..."")  # Next, do wilcoxon tests for continuous and fisher tests for categoricals variables to report p-values in table for # balpos vs negative  # balpos vs negative  # wilcox nonparametric  pcontbalposvsnot_wilcox<-sapply(pnadx_meta_lung[,c("Age", "BMI", "ICUstayduration", "VFD", "SOFAScore", "lips_score", "HR", "SBP", "Temperature", "LowestMAP", "PHa", "WBC", "Platelets", "Creatinine", "CO2", "WorstPFRatio", "TotalRR", "PEEP", "PeakInspiratory", "PlateauPressure", "TVmlperkg")], function(i) wilcox.test(i ~ pnadx_meta_lung$balposbasl)$p.value)  pcontbalposvsnot_wilcox<-data.frame(pcontbalposvsnot_wilcox)  # balpos vs negative categorical - FIsher test  pcatbalposvsnot <-sapply(pnadx_meta_lung[,c("Gender", "HistDiabetes", "HistCOPD", "HistImmunosuppression", "HistCCF", "HistCRF", "IsActiveNeoplasm", "lips_pneumonia", "lips_sepsis", "lips_shock", "lips_aspiration", "Mortality30Day", "Mortality90Day", "AKIFirstWeek")], function(i) fisher.test(i, pnadx_meta_lung$balposbasl)$p.value)  pcatbalposvsnot<-data.frame(pcatbalposvsnot)  ######### ECOLOGICAL ANALYSES  ## Perform basic ecological analyses from taxonomic frequencies at the genus level  ## File with taxonomic and metadata: "pnadx_biom"  # define pathogen and oral bacteria variables from cx pos and cx neg distributions of taxa as explained in the manuscript (based on culture positivity and known composition of the supraglottic pneumotype of the healthy lung microbiome)  # pathogens  # staph  pnadx_biom$staph <- pnadx_biom$`Bacteria;Firmicutes;Bacilli;Bacillales;Staphylococcaceae;Staphylococcus` + pnadx_biom$`Bacteria;Firmicutes;Bacilli;Bacillales;Staphylococcaceae;Staphylococcaceae_unclassified`  # pseudomonas  pnadx_biom$pseudomonas <- pnadx_biom$`Bacteria;Proteobacteria;Gammaproteobacteria;Pseudomonadales;Pseudomonadaceae;Pseudomonas` + pnadx_biom$`Bacteria;Proteobacteria;Gammaproteobacteria;Pseudomonadales;Pseudomonadaceae;Pseudomonadaceae_unclassified`  # Enterobacteriaceae  pnadx_biom$enterobacteriaceae <- pnadx_biom$`Bacteria;Proteobacteria;Gammaproteobacteria;Enterobacteriales;Enterobacteriaceae;Enterobacteriaceae_unclassified`  # escherichia  pnadx_biom$escherichia <- pnadx_biom$`Bacteria;Proteobacteria;Gammaproteobacteria;Enterobacteriales;Enterobacteriaceae;Escherichia-Shigella`  # fusobacterium  pnadx_biom$fusobacterium <- pnadx_biom$`Bacteria;Fusobacteria;Fusobacteriia;Fusobacteriales;Fusobacteriaceae;Fusobacterium`  # haemophilus  pnadx_biom$haemophilus <- pnadx_biom$`Bacteria;Proteobacteria;Gammaproteobacteria;Pasteurellales;Pasteurellaceae;Haemophilus`  # enterococcus  pnadx_biom$enterococcus <- pnadx_biom$`Bacteria;Firmicutes;Bacilli;Lactobacillales;Enterococcaceae;Enterococcus`  # mycoplasma  pnadx_biom$mycoplasma <- pnadx_biom$`Bacteria;Tenericutes;Mollicutes;Mycoplasmatales;Mycoplasmataceae;Mycoplasma`  # create variable contpathogenabund  pnadx_biom <- mutate(pnadx_biom, contpathogenabund = pnadx_biom$staph + pnadx_biom$pseudomonas + pnadx_biom$enterobacteriaceae + pnadx_biom$escherichia + pnadx_biom$fusobacterium + pnadx_biom$haemophilus + pnadx_biom$enterococcus + pnadx_biom$mycoplasma)  # this variable has a break in ~ 50%. Use high anypathogenabund as >50% sequences for any pathogen.  hist(pnadx_biom$contpathogenabund)  ggplot(pnadx_biom, aes(contpathogenabund)) + geom_density()  # anypathogenabund50 = any pathogen added up to >50% abundance  # eachpathogenabund50 = any specific pathogen to dominate >50% of community  pnadx_biom <- mutate (pnadx_biom, anypathogenabund50 = ifelse(pnadx_biom$contpathogenabund>0.5, 1, 0))  pnadx_biom <- mutate(pnadx_biom, eachpathogenabund50 = ifelse(staph >0.5 \| pseudomonas >0.5 \| enterobacteriaceae >0.5 \| haemophilus >0.5 \| escherichia>0.5 \| fusobacterium >0.5 \| enterococcus>0.5 \| mycoplasma>0.5, 1, 0))  pnadx_biom$eachpathogenabund50 <- as.factor(pnadx_biom$eachpathogenabund50)  pnadx_biom$anypathogenabund50 <- as.factor(pnadx_biom$anypathogenabund50)  # ORAL taxa - for definitions of oral taxa as per the Segal Nat Microbiol 2016 paper (supraglottic pneumotype): Prevotella, Rothia, Streptococcus, Veiilonella, Selenomonas, Neisseria, Gemellacea, Paraprevotellacea  #prevotella  pnadx_biom$prevotella <- pnadx_biom$`Bacteria;Bacteroidetes;Bacteroidia;Bacteroidales;Prevotellaceae;Prevotella` + pnadx_biom$`Bacteria;Bacteroidetes;Bacteroidia;Bacteroidales;Prevotellaceae;Prevotella_2` + pnadx_biom$`Bacteria;Bacteroidetes;Bacteroidia;Bacteroidales;Prevotellaceae;Prevotella_6` +  pnadx_biom$`Bacteria;Bacteroidetes;Bacteroidia;Bacteroidales;Prevotellaceae;Prevotella_7`  pnadx_biom$`Bacteria;Bacteroidetes;Bacteroidia;Bacteroidales;Prevotellaceae;Prevotella_9` +  pnadx_biom$`Bacteria;Bacteroidetes;Bacteroidia;Bacteroidales;Prevotellaceae;Prevotellaceae_unclassified`  # Veillonella  pnadx_biom$veillonella <-pnadx_biom$`Bacteria;Firmicutes;Negativicutes;Selenomonadales;Veillonellaceae;Veillonella` + pnadx_biom$`Bacteria;Firmicutes;Negativicutes;Selenomonadales;Veillonellaceae;Veillonellaceae_unclassified`  # gemella  pnadx_biom$gemella <- pnadx_biom$`Bacteria;Firmicutes;Bacilli;Bacillales;Family_XI;Gemella`  # granulicatella  pnadx_biom$granulicattella <- pnadx_biom$`Bacteria;Firmicutes;Bacilli;Lactobacillales;Carnobacteriaceae;Granulicatella`  # rothia  pnadx_biom$rothia <- pnadx_biom$`Bacteria;Actinobacteria;Actinobacteria;Micrococcales;Micrococcaceae;Rothia`  # Neisseria  pnadx_biom$neisseria <- pnadx_biom$`Bacteria;Proteobacteria;Betaproteobacteria;Neisseriales;Neisseriaceae;Neisseria`  # Streptococcus  pnadx_biom$streptococcus <- pnadx_biom$`Bacteria;Firmicutes;Bacilli;Lactobacillales;Streptococcaceae;Streptococcus`  # create variable contoralabund  pnadx_biom <- mutate(pnadx_biom, contoralabund = pnadx_biom$prevotella + pnadx_biom$veillonella + pnadx_biom$rothia + pnadx_biom$neisseria + pnadx_biom$streptococcus + pnadx_biom$granulicattella + pnadx_biom$gemella)  # this variable also has a break in ~ 50%. Use high anyoralabund as >50% sequences for any pathogen.  hist(pnadx_biom$contoralabund)  ggplot(pnadx_biom, aes(contoralabund)) + geom_density()  # anyoralabund50 = any oral taxon added up to >50% abundance  # eachoralabund50 = any specific pathogen to dominate >50% of community  pnadx_biom <- mutate (pnadx_biom, anyoralabund50 = ifelse(pnadx_biom$contoralabund>0.5, 1, 0))  pnadx_biom <- mutate(pnadx_biom, eachoralabund50 = ifelse(prevotella >0.5 \| veillonella >0.5 \| rothia >0.5 \| granulicattella >0.5 \| gemella>0.5 \| streptococcus >0.5 \| neisseria>0.5, 1, 0))  pnadx_biom$anyoralabund50 <- as.factor(pnadx_biom$anyoralabund50)  pnadx_biom$eachoralabund50 <- as.factor(pnadx_biom$eachoralabund50)  # separate lung and oral datasets  pnadx_biom_lung <-filter(pnadx_biom, sampletype=="tracheal")  pnadx_biom_oral <-filter(pnadx_biom, sampletype=="oral")  # examine how pathogen and oral abundance is distributed in the dataset - as expected pathogen and oral are largely reciprocal when looking at them from balposbasl positivity - the other stratification factors did not accomplish such separation  ggplot(pnadx_biom_lung, aes(contpathogenabund)) + geom_density(aes(group=pnadx_biom_lung$balposbasl, fill = pnadx_biom_lung$balposbasl, alpha = 0.2))  ggplot(pnadx_biom, aes(contoralabund)) + geom_density(aes(group=pnadx_biom$balposbasl, fill = pnadx_biom$balposbasl, alpha = 0.2))  # distribution by eachpathogenabund50  ggplot(pnadx_biom, aes(contpathogenabund)) + geom_density(aes(group=pnadx_biom$eachpathogenabund50, fill = pnadx_biom$eachpathogenabund50, alpha = 0.2))  ggplot(pnadx_biom, aes(contoralabund)) + geom_density(aes(group=pnadx_biom$anypathogenabund50, fill = pnadx_biom$anypathogenabund50, alpha = 0.2))  pnadx_biom_lung$balposbasl<- as.factor(pnadx_biom_lung$balposbasl)  # publishable graph for relative pathogen abundance  ggplot(pnadx_biom_lung, aes(contpathogenabund)) + geom_density(aes(group=pnadx_biom_lung$balposbasl, fill = pnadx_biom_lung$balposbasl, alpha = 0.2)) + xlab("Relative Pathogen Abundance") + theme(legend.position = 'none', axis.text.x = element_text(size=24, face="bold"), axis.text.y = element_text(face="bold", size = 12), axis.title.y = element_text(size=18), title =element_text(size=30, face='bold')) + scale_fill_manual(values=c("turquoise", "red")) + geom_text(size=14, aes(0.78, 4, label="Cx positive", colour="red")) + geom_text(size = 14, aes(0.23, 4.5, label="Cx negative", colour="turquoise"))  # publishable graph for relative oral abundance  ggplot(pnadx_biom_lung, aes(contoralabund)) + geom_density(aes(group=pnadx_biom_lung$balposbasl, fill = pnadx_biom_lung$balposbasl, alpha = 0.2)) + xlab("Relative Oral Taxa Abundance") + theme(legend.position = 'none', axis.text.x = element_text(size=24, face="bold"), axis.text.y = element_text(face="bold", size = 12), axis.title.y = element_text(size=18), title =element_text(size=30, face='bold')) + scale_fill_manual(values=c("turquoise", "red")) + geom_text(size=14, aes(0.25, 4.2, label="Cx positive", colour="red")) + geom_text(size = 14, aes(0.7, 2, label="Cx negative", colour="turquoise"))  #### Beta diversity analyses with vegan  # Use the taxa table: "ALIR_taxa_genus_summary_table_clean"  # perform beta-diversity analyses in vegan for oral/lung by pneumonia vs not and by cx positivity vs not  #filter for samples in the pnadx dataset  pnadx_vegan <- subset(ALIR_taxa_genus_summary_table_clean, select = -Total)  pnadx_vegan <- filter(pnadx_vegan, Sample_ID %in% pnadx_meta_SampleIDs)  #sort by Sample_ID variable and confirm they are equal  pnadx_meta <- arrange(pnadx_meta, Sample_ID)  pnadx_vegan <- arrange(pnadx_vegan, Sample_ID)  pnadx_vegan$Sample_ID  pnadx_meta$Sample_ID # confirmed that these are aligned properly.  # now remove Sample_ID from taxa table to ensure all are numeric  pnadx_vegan<- subset(pnadx_vegan, select = -Sample_ID)  # create new factor of balcx positivity and site  pnadx_meta$balposbasl[is.na(pnadx_meta$balposbasl)]<- 0  pnadx_meta <- mutate(pnadx_meta, balcx_site = ifelse(pnadx_meta$balposbasl==1 & pnadx_meta$sampletype=="tracheal", "lungcxpos", NA))  pnadx_meta <- mutate(pnadx_meta, balcx_site = ifelse(pnadx_meta$balposbasl==1 & pnadx_meta$sampletype=="oral", "oralcxpos", pnadx_meta$balcx_site))  pnadx_meta <- mutate(pnadx_meta, balcx_site = ifelse(pnadx_meta$balposbasl==0 & pnadx_meta$sampletype=="oral", "oralcxneg", pnadx_meta$balcx_site))  pnadx_meta <- mutate(pnadx_meta, balcx_site = ifelse(pnadx_meta$balposbasl==0 & pnadx_meta$sampletype=="tracheal", "lungcxneg", pnadx_meta$balcx_site))  pnadx_meta$balcx_site <-as.factor(pnadx_meta$balcx_site)  # balcx_site  pnadx_vegan_mds <- metaMDS(pnadx_vegan, noshare =TRUE, k = 3) # stress 0.17  # define factor for ordination plot  fac <- pnadx_meta$balcx_site  # NMDS plot for balcx_site  plot(pnadx_vegan_mds, disp="sites", type = "n", xlim=c(-0.9, 0.9), ylim=c(-0.9, 0.9))  points(pnadx_vegan_mds, disp="sites", pch=21, col = "#FF0000", cex = 2, select=which(fac=="lungcxpos"))  points(pnadx_vegan_mds, disp="sites", pch=24, col = "#FF0000", cex = 2, select=which(fac=="oralcxpos"))  points(pnadx_vegan_mds, disp="sites", pch=24, col = "#3399FF", cex = 2, select=which(fac=="oralcxneg"))  points(pnadx_vegan_mds, disp="sites", pch=21, col = "#3399FF", cex = 2, select=which(fac=="lungcxneg"))  ordiellipse(pnadx_vegan_mds, group=fac, show.groups="lungcxpos", col = "#FF0000", lwd = 2)  ordiellipse(pnadx_vegan_mds, group=fac, show.groups="oralcxpos", col = "#FF0000", lwd = 2)  ordiellipse(pnadx_vegan_mds, group=fac, show.groups="oralcxneg", col = "#3399FF", lwd = 2)  ordiellipse(pnadx_vegan_mds, group=fac, show.groups="lungcxneg", col = "#3399FF", lwd = 2)  ordispider(pnadx_vegan_mds, group=fac, show.groups="oralcxpos", col = "#FF0000", label = TRUE)  ordispider(pnadx_vegan_mds, group=fac, show.groups="oralcxneg", col = "#CCCCCC", label = TRUE)  ordispider(pnadx_vegan_mds, group=fac, show.groups="lungcxneg", col = "#CCCCCC", label = TRUE)  ordispider(pnadx_vegan_mds, group=fac, show.groups="lungcxpos", col = "#FF0000", label = TRUE)  # adonis  betadivbybalcx_site <- betadiver(pnadx_vegan, "z")  adonis(betadivbybalcx_site ~ balcx_site, pnadx_meta, perm=1000)  # Df SumsOfSqs MeanSqs F.Model R2 Pr(>F)  # balcx_site 3 2.0614 0.68714 2.8393 0.07438 0.000999 ***.06368 0.000999 ***  ### vegan analyses for lung samples only  #filter for samples in the pnadx dataset  pnadx_vegan_lung <- subset(ALIR_taxa_genus_summary_table_clean, select = -Total)  pnadx_meta_lung_sampleids <- pnadx_meta_lung$Sample_ID  pnadx_vegan_lung <- filter(pnadx_vegan_lung, Sample_ID %in% pnadx_meta_lung_sampleids)  #sort by Sample_ID variable and confirm they are equal  pnadx_vegan_lung <- arrange(pnadx_vegan_lung, Sample_ID)  pnadx_meta_lung <- arrange(pnadx_meta_lung, Sample_ID)  pnadx_meta_lung$Sample_ID  pnadx_vegan_lung$Sample_ID # confirmed that these are aligned properly.  # now remove Sample_ID from taxa table to ensure all are numeric  pnadx_vegan_lung<- subset(pnadx_vegan_lung, select = -Sample_ID)  # BAL pos  pnadx_meta_lung$balposbasl <-as.character(pnadx_meta_lung$balposbasl)  pnadx_meta_lung$balposbasl[pnadx_meta_lung$balposbasl=="0"] <- "RespCxNeg"  pnadx_meta_lung$balposbasl[pnadx_meta_lung$balposbasl=="1"] <- "RespCxPos"  pnadx_meta_lung$balposbasl <-as.factor(pnadx_meta_lung$balposbasl)  # define factor for ordination plot  fac <- pnadx_meta_lung$balposbasl  attach(pnadx_meta_lung)  # NMDS plot for BALPOS  plot(pnadx_pneumonia_mds, disp="sites", type = "n", xlim=c(-1.2, 1.2), ylim=c(-1.2, 1.2))  points(pnadx_pneumonia_mds, disp="sites", pch=21, col = "#FF3333", cex = 2, select=which(fac=="RespCxPos"))  points(pnadx_pneumonia_mds, disp="sites", pch=24, col = "#0099FF", cex = 2, select=which(fac=="RespCxNeg"))  ordiellipse(pnadx_pneumonia_mds, group=fac, show.groups="RespCxPos", col = "#FF3333", lwd = 2)  ordiellipse(pnadx_pneumonia_mds, group=fac, show.groups="RespCxNeg", col = "#0099FF", lwd = 2)  ordispider(pnadx_pneumonia_mds, group=fac, show.groups="RespCxNeg", col = "#0099FF", label = TRUE)  ordispider(pnadx_pneumonia_mds, group=fac, show.groups="RespCxPos", col = "#FF3333", label = TRUE)  # perform permanova based on the variable balpos for formal comparison  betadivbybalpos <- betadiver(pnadx_vegan_lung, "z")  adonis(betadivbybalpos~balposbasl, pnadx_meta_lung, perm=1000)  # Df SumsOfSqs MeanSqs F.Model R2 Pr(>F)  # balposbasl 1 0.7047 0.70474 2.7197 0.04795 0.002997 **  # vegan analyses for oral only  pnadx_vegan_oral <- subset(ALIR_taxa_genus_summary_table_clean, select = -Total)  pnadx_meta_oral <- filter(pnadx_meta, sampletype=="oral")  pnadx_meta_oral_sampleids <- pnadx_meta_oral$Sample_ID  pnadx_vegan_oral <- filter(pnadx_vegan_oral, Sample_ID %in% pnadx_meta_oral_sampleids)  #sort by Sample_ID variable and confirm they are equal  pnadx_vegan_oral <- arrange(pnadx_vegan_oral, Sample_ID)  pnadx_meta_oral <- arrange(pnadx_meta_oral, Sample_ID)  pnadx_meta_oral$Sample_ID  pnadx_vegan_oral$Sample_ID # confirmed that these are aligned properly.  # now remove Sample_ID from taxa table to ensure all are numeric  pnadx_vegan_oral<- subset(pnadx_vegan_oral, select = -Sample_ID)  # BAL pos  pnadx_meta_oral$balposbasl <-as.character(pnadx_meta_oral$balposbasl)  pnadx_meta_oral$balposbasl[pnadx_meta_oral$balposbasl=="0"] <- "RespCxNeg"  pnadx_meta_oral$balposbasl[pnadx_meta_oral$balposbasl=="1"] <- "RespCxPos"  pnadx_meta_oral$balposbasl <-as.factor(pnadx_meta_oral$balposbasl)  # define factor for ordination plot  fac <- pnadx_meta_oral$balposbasl  # NMDS plot for BALPOS  plot(pnadx_oral_mds, disp="sites", type = "n", xlim=c(-1.2, 1.2), ylim=c(-1.2, 1.2))  points(pnadx_oral_mds, disp="sites", pch=21, col = "#FF3333", cex = 2, select=which(fac=="RespCxPos"))  points(pnadx_oral_mds, disp="sites", pch=24, col = "#0099FF", cex = 2, select=which(fac=="RespCxNeg"))  ordiellipse(pnadx_oral_mds, group=fac, show.groups="RespCxPos", col = "#FF3333", lwd = 2)  ordiellipse(pnadx_oral_mds, group=fac, show.groups="RespCxNeg", col = "#0099FF", lwd = 2)  ordispider(pnadx_oral_mds, group=fac, show.groups="RespCxNeg", col = "#0099FF", label = TRUE)  ordispider(pnadx_oral_mds, group=fac, show.groups="RespCxPos", col = "#FF3333", label = TRUE)  # perform permanova based on the variable balpos for formal comparison  betadivbybalpos <- betadiver(pnadx_vegan_oral, "z")  adonis(betadivbybalpos~balposbasl, pnadx_meta_oral, perm=1000)  # Df SumsOfSqs MeanSqs F.Model R2 Pr(>F)  # balposbasl 1 0.7113 0.71130 3.1721 0.0575 0.000999 ***  # impressive difference in oral communities.  #### Alpha diversity analyses  # alpha diversity analyses  ###### create publishable graphs for alpha diversity comparisons between pneumonia and balcxpos  pnadx_alphadivgraphdataset_lung <- subset(pnadx_meta_lung, select = c(Sample_ID, shannon, dominance, lips_aspiration, balposbasl, lips_pneumonia, cxposbasl))  # make long format to enable simultaneous presentation of shannon and dominance  library(tidyr)  pnadx_alphadivgraphdataset_lung_long <- gather(pnadx_alphadivgraphdataset_lung, alphadiv, measurement, shannon:dominance, factor_key = TRUE)  View(pnadx_alphadivgraphdataset_lung_long)  # balpos  ggplot(pnadx_alphadivgraphdataset_lung_long, aes(x = balposbasl, y = measurement, fill = balposbasl)) +  geom_boxplot(lwd=0.8, outlier.shape = 8) +  geom_jitter(width = 0.1, size = 8, shape = 1) +  facet_wrap(~alphadiv, scales = "free_y") +  ylab("alpha diversity metric") +  xlab("Respiratory Cultures") +  theme(legend.position = 'none', axis.text.x = element_text(size=20, face="bold"), axis.text.y = element_text(face="bold", size = 16), axis.title.y = element_text(size=14), title =element_text(size=30, face='bold')) +  theme(strip.text = element_text(face="bold", size = 20, colour = "blue")) +  theme(panel.background = element_rect(fill = "white"), panel.grid.major = element_line(size = 0.5, linetype = 'solid', colour="gray")) + scale_fill_manual(values = c("#0099FF", "#CC0000")) +  scale_x_discrete(labels = c("Negative", "Positive"))  # statistical testing for shannon and dominance by balposbasl  wilcox.test(pnadx_meta_lung$shannon ~ pnadx_meta_lung$balposbasl) # 0.02168  wilcox.test(pnadx_meta_lung$dominance ~ pnadx_meta_lung$balposbasl) # 0.0413  #### ################# Taxonomic composition in culture positive and negative cases  # define "other" abundance in biom file  pnadx_biom_lung$other <- 1- (pnadx_biom_lung$contpathogenabund + pnadx_biom_lung$contoralabund)  pnadx_biom_lung  pnadx_biom_lung$balposbasl[is.na(pnadx_biom_lung$balposbasl)] <-0  pnadx_biom_lung$cxposbasl [is.na(pnadx_biom_lung$cxposbasl)] <-0  pnadx_biom_lung$bloodcxposbasl[is.na(pnadx_biom_lung$bloodcxposbasl)] <-0  pnadx_biom_lung$balposbasl <- as.factor(pnadx_biom_lung$balposbasl)  # filter balpos and negative files  pnadx_biom_lung_balpos <- filter(pnadx_biom_lung, balposbasl==1)  pnadx_biom_lung_balneg <- filter(pnadx_biom_lung, balposbasl==0)  # select only relevant taxa plus other  pnadx_biom_lung_balpos_taxa <- subset(pnadx_biom_lung_balpos, select = c(Sample_ID, staph , pseudomonas, enterobacteriaceae , escherichia , fusobacterium , haemophilus , enterococcus , mycoplasma, prevotella , veillonella , rothia, neisseria, streptococcus, granulicattella, gemella, other))  # transpose dataset  ## https://stackoverflow.com/questions/7970179/transposing-a-dataframe-maintaining-the-first-column-as-heading  # https://stackoverflow.com/questions/29511215/convert-row-names-into-first-column  pnadx_biom_lung_balpos_taxa_transposed <- as.data.frame(t(pnadx_biom_lung_balpos_taxa[, -1]))  colnames(pnadx_biom_lung_balpos_taxa_transposed) <- pnadx_biom_lung_balpos_taxa$Sample_ID  pnadx_biom_lung_balpos_taxa_transposed$taxon <- rownames(pnadx_biom_lung_balpos_taxa_transposed)  # melt dataset for visualizations  lungbalpostaxa <- melt(pnadx_biom_lung_balpos_taxa_transposed, id.vars = "taxon", variable.name = "SampleID", value.name = "abundance")  lungbalpostaxa$Taxa <- as.factor(lungbalpostaxa$taxon)  ggplot(lungbalpostaxa, aes(x=SampleID, y = abundance, fill = Taxa)) + geom_bar(stat="identity", colour = "black")  # reorder taxa  lungbalpostaxa$Taxa <- ordered(lungbalpostaxa$Taxa , levels = c("staph", "pseudomonas", "enterobacteriaceae" , "escherichia", "fusobacterium" , "haemophilus", "enterococcus", "mycoplasma", "prevotella", "veillonella", "rothia", "neisseria", "streptococcus", "granulicattella", "gemella", "other"))  ggplot(lungbalpostaxa, aes(x=SampleID, y = abundance, fill = Taxa)) + geom_bar(stat="identity") + theme_classic()  # create color pallette  # http://www.cookbook-r.com/Graphs/Colors_(ggplot2)/  colorpalette_lungbaltaxa <- c("#990066","#FF0066", "#FF0000", "#FF6666", "#CC6666", "#990000", "#996666", "#FF6633",  "#3399FF", "#0066CC", "#003366", "#66CCFF", "#00CCFF", "#00FFFF", "#CCFFFF",  "#CCCCCC")  # reorder observations in trach_balpos_taxa dataset to have concordant cases first and discordant later  lungbalpostaxa_order <- c("0102.1031.1.T", "0102.3769.1.T", "0102.3995.1.T", "0102.3699.1.T.N", "0102.47.1.T", "0102.3476.1.T", "0102.3508.1.T", "0102.3673.1.T", "0102.3772.1.T", "0102.3931.3.T.2", "0102.3734.1.T", "0102.3902.1.T")  library(gdata)  # http://stackoverflow.com/questions/11977102/order-data-frame-rows-according-to-a-target-vector-that-specifies-the-desired-or  lungbalpostaxa$SampleID <- reorder.factor(lungbalpostaxa$SampleID, new.order = lungbalpostaxa_order)  # optimize label with italics/capitals for taxa names.  lungbalpostaxa_2 <- lungbalpostaxa  lungbalpostaxa_2$Taxa <- as.character(lungbalpostaxa_2$Taxa)  lungbalpostaxa_2$Taxa[lungbalpostaxa_2$Taxa=="staph"] <- "Staphylococcus"  lungbalpostaxa_2$Taxa[lungbalpostaxa_2$Taxa=="pseudomonas"] <- "Pseudomonas"  lungbalpostaxa_2$Taxa[lungbalpostaxa_2$Taxa=="enterobacteriaceae"] <- "Enterobacteriaceae"  lungbalpostaxa_2$Taxa[lungbalpostaxa_2$Taxa=="escherichia"] <- "Escherichia"  lungbalpostaxa_2$Taxa[lungbalpostaxa_2$Taxa=="fusobacterium"] <- "Fusobacterium"  lungbalpostaxa_2$Taxa[lungbalpostaxa_2$Taxa=="haemophilus"] <- "Haemophilus"  lungbalpostaxa_2$Taxa[lungbalpostaxa_2$Taxa=="enterococcus"] <- "Enterococcus"  lungbalpostaxa_2$Taxa[lungbalpostaxa_2$Taxa=="mycoplasma"] <- "Mycoplasma"  lungbalpostaxa_2$Taxa[lungbalpostaxa_2$Taxa=="prevotella"] <- "Prevotella"  lungbalpostaxa_2$Taxa[lungbalpostaxa_2$Taxa=="veillonella"] <- "Veillonella"  lungbalpostaxa_2$Taxa[lungbalpostaxa_2$Taxa=="rothia"] <- "Rothia"  lungbalpostaxa_2$Taxa[lungbalpostaxa_2$Taxa=="neisseria"] <- "Neisseria"  lungbalpostaxa_2$Taxa[lungbalpostaxa_2$Taxa=="streptococcus"] <- "Streptococcus"  lungbalpostaxa_2$Taxa[lungbalpostaxa_2$Taxa=="granulicattella"] <- "Granulicattella"  lungbalpostaxa_2$Taxa[lungbalpostaxa_2$Taxa=="gemella"] <- "Gemella"  lungbalpostaxa_2$Taxa[lungbalpostaxa_2$Taxa=="other"] <- "Other"  lungbalpostaxa_2$Taxa <- as.factor(lungbalpostaxa_2$Taxa)  View(lungbalpostaxa_2)  lungbalpostaxa_2$Taxa <- ordered(lungbalpostaxa_2$Taxa , levels = c("Staphylococcus", "Pseudomonas", "Enterobacteriaceae" , "Escherichia", "Fusobacterium" , "Haemophilus", "Enterococcus", "Mycoplasma", "Prevotella", "Veillonella", "Rothia", "Neisseria", "Streptococcus", "Granulicattella", "Gemella", "Other"))  ggplot(lungbalpostaxa_2, aes(x=SampleID, y = abundance, fill = Taxa)) + geom_bar(stat="identity", colour = "black") +  scale_fill_manual(breaks = c("Staphylococcus", "Pseudomonas", "Enterobacteriaceae" , "Escherichia", "Fusobacterium" , "Haemophilus", "Enterococcus", "Mycoplasma", "Prevotella", "Veillonella", "Rothia", "Neisseria", "Streptococcus", "Granulicattella", "Gemella", "Other"), values = colorpalette_lungbaltaxa) +  ggtitle("Taxonomic composition for culture positive samples") +  scale_x_discrete(breaks = NULL) + xlab("") + ylab("Relative Abundance") +  theme(legend.position = 'bottom', axis.text.y = element_text(face="bold")) +  theme(strip.background = element_blank(), strip.text = element_blank(), title = element_text(size=14, face='bold'), legend.text = element_text(size=14, face="italic")) +  geom_text(aes(1, 0.5, label = "S.aureus", angle = 90), size = 8, colour = "black") +  geom_text(aes(2, 0.6, label = "S.aureus", angle = 90), size = 8, colour = "black") +  geom_text(aes(3, 0.7, label = "S.aureus", angle = 90), size = 8, colour = "black") +  geom_text(aes(4, 0.8, label = "S.aureus", angle = 90), size = 6, colour = "black") +  geom_text(aes(5, 0.9, label = "S.aureus", angle = 90), size = 3, colour = "black") +  geom_text(aes(5, 0.5, label = "H. Flu", angle = 90), size = 6, colour = "black") +  geom_text(aes(6, 0.6, label = "Pseudomonas", angle = 90), size = 7, colour = "black") +  geom_text(aes(7, 0.5, label = "Klebsiella", angle = 90), size = 7, colour = "black") +  geom_text(aes(8, 0.5, label = "Klebsiella", angle = 90), size = 7, colour = "black") +  geom_text(aes(9, 0.5, label = "E.coli", angle = 90), size = 7, colour = "black") +  geom_text(aes(10, 0.87, label = "Klebsiella", angle = 90), size = 4, colour = "black") +  geom_text(aes(11, 0.98, label = "Staph"), size = 4, colour = "black") +  geom_text(aes(10, 0.5, label = "-"), size = 15, colour = "black") +  geom_text(aes(11, 0.5, label = "-"), size = 15, colour = "black") +  geom_text(aes(12, 0.5, label = "-"), size = 15, colour = "black") +  geom_segment(aes(x=0.5, y=-0.05, xend=9, yend=-0.05)) +  geom_segment(aes(x=10, y=-0.08, xend=12.5, yend=-0.08), linetype = "dashed") +  geom_text(aes(3, -0.08, label = "Culture concordance"), size = 8, colour = "black") +  geom_text(aes(11, -0.10, label = "Culture discordance"), size = 5, colour = "black") +  geom_text(aes(1, 1.04, label = "1"), size = 4, colour = "blue") +  geom_text(aes(2, 1.04, label = "2"), size = 4, colour = "blue") +  geom_text(aes(3, 1.04, label = "3"), size = 4, colour = "blue") +  geom_text(aes(4, 1.04, label = "4"), size = 4, colour = "blue") +  geom_text(aes(5, 1.04, label = "5"), size = 4, colour = "blue") +  geom_text(aes(6, 1.04, label = "6"), size = 4, colour = "blue") +  geom_text(aes(7, 1.04, label = "7"), size = 4, colour = "blue") +  geom_text(aes(8, 1.04, label = "8"), size = 4, colour = "blue") +  geom_text(aes(9, 1.04, label = "9"), size = 4, colour = "blue") +  geom_text(aes(10, 1.04, label = "10"), size = 4, colour = "blue") +  geom_text(aes(11, 1.04, label = "11"), size = 4, colour = "blue") +  geom_text(aes(12, 1.04, label = "12"), size = 4, colour = "blue") +  geom_text(aes(5.3, 1.04, label="#"), size=4, colour = "black") +  geom_text(aes(11.3, 1.04, label="#"), size=4, colour = "black")      ####################### taxonomic composition of balneg samples  # select only relevant taxa plus other  pnadx_biom_lung_balneg_taxa <- subset(pnadx_biom_lung_balneg, select = c(Sample_ID, staph , pseudomonas, enterobacteriaceae , escherichia , fusobacterium , haemophilus , enterococcus , mycoplasma, prevotella , veillonella , rothia, neisseria, streptococcus, granulicattella, gemella, other))  # remove sample 0102.3818.1.T  pnadx_biom_lung_balneg_taxa <- pnadx_biom_lung_balneg_taxa[-23, ]  # transpose dataset  ## https://stackoverflow.com/questions/7970179/transposing-a-dataframe-maintaining-the-first-column-as-heading  # https://stackoverflow.com/questions/29511215/convert-row-names-into-first-column  pnadx_biom_lung_balneg_taxa_transposed <- as.data.frame(t(pnadx_biom_lung_balneg_taxa[, -1]))  colnames(pnadx_biom_lung_balneg_taxa_transposed) <- pnadx_biom_lung_balneg_taxa$Sample_ID  pnadx_biom_lung_balneg_taxa_transposed$taxon <- rownames(pnadx_biom_lung_balneg_taxa_transposed)  # melt dataset for visualizations  lungbalnegtaxa <- melt(pnadx_biom_lung_balneg_taxa_transposed, id.vars = "taxon", variable.name = "SampleID", value.name = "abundance")  lungbalnegtaxa$Taxa <- as.factor(lungbalnegtaxa$taxon)  ggplot(lungbalnegtaxa, aes(x=SampleID, y = abundance, fill = Taxa)) + geom_bar(stat="identity", colour = "black")  # reorder taxa  lungbalnegtaxa$Taxa <- ordered(lungbalnegtaxa$Taxa , levels = c("staph", "pseudomonas", "enterobacteriaceae" , "escherichia", "fusobacterium" , "haemophilus", "enterococcus", "mycoplasma", "prevotella", "veillonella", "rothia", "neisseria", "streptococcus", "granulicattella", "gemella", "other"))  # reorder SampleIDs  lungbalnegtaxa_order <- c("0102.1819.1.T.N", "0102.3964.1.T", "0102.3927.1.T.2", "0102.3935.1.T" , "0102.3966.1.T.2", "0102.3928.1.T", "0102.3987.1.T", "0102.3774.1.T", "0102.3862.1.T", "0102.3904.1.T", "0102.1357.1.T.N", "0102.1767.1.T", "0102.1971.1.T", "0102.3189.1.T.N", "0102.3538.1.T", "0102.3641.1.T", "0102.3644.1.T", "0102.3645.1.T", "0102.3646.1.T", "0102.3672.1.T", "0102.3731.1.T", "0102.3732.1.T", "0102.3747.1R.T", "0102.3768.1.T", "0102.3770.1.T", "0102.3771.1.T", "0102.3775.1.T", "0102.3801.1.T", "0102.3838.1.T", "0102.3847.1.T", "0102.3864.1.T","0102.3497.3.T", "0102.3901.1.T", "0102.3930.1.T", "0102.3986.1.T.2", "0102.3932.1.T", "0102.3933.1.T", "0102.3996.1.T", "0102.3934.1.T", "0102.4048.1.T", "0102.4055.1.T", "0102.3881.1.T.2")  library(gdata)  # http://stackoverflow.com/questions/11977102/order-data-frame-rows-according-to-a-target-vector-that-specifies-the-desired-or  lungbalnegtaxa$SampleID <- reorder.factor(lungbalnegtaxa$SampleID, new.order = lungbalnegtaxa_order)  # graph for culture negative samples#################################  ggplot(lungbalnegtaxa, aes(x=SampleID, y = abundance, fill = Taxa)) +  geom_bar(stat="identity", colour = "black") +  scale_fill_manual(values = colorpalette_lungbaltaxa) +  scale_x_discrete(breaks = NULL) +  ggtitle("Taxonomic composition for culture negative samples") + xlab("") + ylab("Relative Abundance") +  theme(legend.position = 'bottom',axis.text.y = element_text(face="bold"), legend.text = element_text(size=15, face="bold")) + theme(strip.background = element_blank(), strip.text = element_blank(), title = element_text(size=14, face='bold')) +  geom_segment(aes(x=0.5, y=-0.03, xend=9, yend=-0.03), size = 2, linetype = "solid", colour = "red") +  geom_segment(aes(x=10, y=-0.04, xend=44, yend=-0.04), size = 2, linetype = "longdash", colour = "blue") +  geom_text(aes(4.1, -0.08, label = "Missed pathogens"), size = 5, colour = "red") +  geom_text(aes(25, -0.08, label = "Oral taxa abundance"), size = 8, colour = "blue")  # graph without legend to combine with balposbasl  # graph for culture negative samples#################################  ggplot(lungbalnegtaxa, aes(x=SampleID, y = abundance, fill = Taxa)) +  geom_bar(stat="identity", colour = "black") +  scale_fill_manual(values = colorpalette_lungbaltaxa) +  scale_x_discrete(breaks = NULL) +  ggtitle("Taxonomic composition for culture negative samples") + xlab("") + ylab("Relative Abundance") +  theme(legend.position = 'none',axis.text.y = element_text(face="bold"), legend.text = element_text(size=15, face="bold")) + theme(strip.background = element_blank(), strip.text = element_blank(), title = element_text(size=14, face='bold')) +  geom_segment(aes(x=0.5, y=-0.03, xend=9, yend=-0.03), size = 2, linetype = "solid", colour = "red") +  geom_segment(aes(x=10, y=-0.04, xend=44, yend=-0.04), size = 2, linetype = "longdash", colour = "blue") +  geom_text(aes(5, -0.08, label = "Missed pathogens"), size = 5, colour = "red") +  geom_text(aes(25, -0.08, label = "Oral taxa abundance"), size = 8, colour = "blue") +  geom_text(aes(14.1, 1.03, label="#"), size=4, colour = "black") +  geom_text(aes(22.1, 1.03, label="#"), size=4, colour = "black") +  geom_text(aes(23.1, 1.03, label="#"), size=4, colour = "black") +  geom_text(aes(26.1, 1.03, label="#"), size=4, colour = "black")      ####### diagnostic framework for eachpathogen abund 50.  # remove obs 31 from pnadx_biom_lung as this was removed at clean up phase in qiime  pnadx_biom_lung <- pnadx_biom_lung[-31, ]  oddsratio.fisher(pnadx_biom_lung$eachpathogenabund50, pnadx_biom_lung$balposbasl) # OR 20.2 (3.4-224.3), p<10-5.  oddsratio.fisher(pnadx_biom_lung$anypathogenabund50, pnadx_biom_lung$balposbasl) # OR 38.2 (4.5-1817.6), p<10-5.  # look at negative associations with oral taxa abundance  oddsratio.small(pnadx_biom_lung$eachoralabund50, pnadx_biom_lung$balposbasl) # highly statistically significant  oddsratio.small(pnadx_biom_lung$anyoralabund50, pnadx_biom_lung$balposbasl) # highly statistically significant and clinically more relevant, indicating that if >50% of the community is occupied by oral taxa, then chances of a positive culture are extremely low  # OR 0.08 0.0007744463 0.2586769. p<10-6    #biomarker graphs  # use the facet_wrap function to create nice publishable graphs, but this needs a gather function of the dataset.  # http://www.sthda.com/english/wiki/tidyr-crucial-step-reshaping-data-with-r-for-easier-analyses  pnadx_4biomarkergraphs <- subset(pnadx_biom_lung, select = c(subjectid, grouplips, balposbasl,eachpathogenabund50, anypathogenabund50, il6_rd, rage_rd, tnfr1_rd ,il8_rd, ))  # change variable names to make them look nice for graph.  colnames(pnadx_4biomarkergraphs)[colnames(pnadx_4biomarkergraphs)=='il6_rd'] <- 'IL-6'  colnames(pnadx_4biomarkergraphs)[colnames(pnadx_4biomarkergraphs)=='rage_rd'] <- 'RAGE'  colnames(pnadx_4biomarkergraphs)[colnames(pnadx_4biomarkergraphs)=='tnfr1_rd'] <- 'TNFR1'  colnames(pnadx_4biomarkergraphs)[colnames(pnadx_4biomarkergraphs)=='il8_rd'] <- 'IL-8'  library(tidyr)  # convert to long inorder to graph cytokine variable  pnadx_4biomarkergraphs_long <- gather(pnadx_4biomarkergraphs, cytokine, value,`IL-10`:`PCT`, factor_key = TRUE)  View(pnadx_4biomarkergraphs_long)  # Graph:  ggplot(pnadx_4biomarkergraphs_long, aes(x = eachpathogenabund50, y = value, fill = eachpathogenabund50), show.legend=F) +  geom_boxplot(lwd=0.8, notch = FALSE, aes(group=pnadx_4biomarkergraphs_long$eachpathogenabund50), alpha=0.3) +  geom_jitter(width = 0.1, size=4, aes(shape = factor(pnadx_4biomarkergraphs_long$balposbasl)), show.legend=F) +  facet_wrap(~cytokine, scales = "free_y") +  scale_shape_manual(values = c(1, 16)) +  theme(legend.position = 'none') +  xlab("Pathogen Abundance") +  ylab("biomarker log-concentrations (pg/ml)") +  theme(legend.position = 'none', axis.text.x = element_text(size=20, face="bold"), axis.text.y = element_text(face="bold", size = 12), axis.title.y = element_text(size=14), title =element_text(size=20, face='bold')) +  theme(panel.background = element_rect(fill = "white"), panel.grid.major = element_line(size = 0.5, linetype = 'dashed', colour="gray"), strip.text = element_text(face="bold", size = 20, colour = "black")) +  scale_x_discrete(labels = c("< 50%", "> 50%")) +  scale_fill_manual(values = c( "#CCFFFF", "#FFCCCC")) +  scale_colour_manual(values=c("black", "red", "blue"))  # examine associations of biomarkers with pathogen abundance >50%  # examine independence of biomarker association to culture positivity  # entire cohort  # il6  fit <- lm(il6_rd ~ eachpathogenabund50, data=pnadx_biom_lung)  summary(fit)  fit <- lm(il6_rd ~ eachpathogenabund50 + balposbasl, data=pnadx_biom_lung)  summary(fit)  # rage  fit <- lm(rage_rd~ eachpathogenabund50, data=pnadx_biom_lung)  summary(fit)  fit <- lm(rage_rd~ eachpathogenabund50 + balposbasl, data=pnadx_biom_lung)  summary(fit)  # tnfr1  fit <- lm(tnfr1_rd~ eachpathogenabund50, data=pnadx_biom_lung)  summary(fit)  fit <- lm(tnfr1_rd~ eachpathogenabund50 + balposbasl, data=pnadx_biom_lung)  summary(fit)  # il8  fit <- lm(il8_rd~ eachpathogenabund50, data=pnadx_biom_lung)  summary(fit)  fit <- lm(il8_rd~ eachpathogenabund50 + balposbasl, data=pnadx_biom_lung)  summary(fit)  # procalcitonin  fit <- lm(procalcitonin_rd~ eachpathogenabund50, data=pnadx_biom_lung)  summary(fit)  fit <- lm(procalcitonin_rd~ eachpathogenabund50 + balposbasl, data=pnadx_biom_lung)  summary(fit) |
| --- |

Bibliography

Aliferis, C. F., Statnikov, A., Tsamardinos, I., Mani, S., and Koutsoukos, X. D. (2010a). Local Causal and Markov Blanket Induction for Causal Discovery and Feature Selection for Classification Part I: Algorithms and Empirical Evaluation. *Journal of Machine Learning Research*.

Aliferis, C. F., Statnikov, A., Tsamardinos, I., Mani, S., and Koutsoukos, X. D. (2010b). Local Causal and Markov Blanket Induction for Causal Discovery and Feature Selection for Classification Part II: Analysis and Extensions. *Journal of Machine Learning Research*.

ARDS Definition Task Force, Ranieri, V. M., Rubenfeld, G. D., Thompson, B. T., Ferguson, N. D., Caldwell, E., Fan, E., Camporota, L., and Slutsky, A. S. (2012). Acute respiratory distress syndrome: the Berlin Definition. *JAMA* 307, 2526–2533. doi:10.1001/jama.2012.5669.

Calfee, C. S., Delucchi, K., Parsons, P. E., Thompson, B. T., Ware, L. B., Matthay, M. A., and NHLBI ARDS Network (2014). Subphenotypes in acute respiratory distress syndrome: latent class analysis of data from two randomised controlled trials. *Lancet Respir. Med.* 2, 611–620. doi:10.1016/S2213-2600(14)70097-9.

Caporaso, J. G., Kuczynski, J., Stombaugh, J., Bittinger, K., Bushman, F. D., Costello, E. K., Fierer, N., Peña, A. G., Goodrich, J. K., Gordon, J. I., et al. (2010). QIIME allows analysis of high-throughput community sequencing data. *Nat Methods* 7, 335–336. doi:10.1038/nmeth.f.303.

Caporaso, J. G., Lauber, C. L., Walters, W. A., Berg-Lyons, D., Huntley, J., Fierer, N., Owens, S. M., Betley, J., Fraser, L., Bauer, M., et al. (2012). Ultra-high-throughput microbial community analysis on the Illumina HiSeq and MiSeq platforms. *ISME J* 6, 1621–1624. doi:10.1038/ismej.2012.8.

Dixon, P. (2003). VEGAN, a package of R functions for community ecology. *Journal of Vegetation Science* 14, 927–930. doi:10.1111/j.1654-1103.2003.tb02228.x.

Famous, K. R., Delucchi, K., Ware, L. B., Kangelaris, K. N., Liu, K. D., Thompson, B. T., Calfee, C. S., and ARDS Network (2017). Acute respiratory distress syndrome subphenotypes respond differently to randomized fluid management strategy. *Am J Respir Crit Care Med* 195, 331–338. doi:10.1164/rccm.201603-0645OC.

Gong, M. N., Thompson, B. T., Williams, P., Pothier, L., Boyce, P. D., and Christiani, D. C. (2005). Clinical predictors of and mortality in acute respiratory distress syndrome: potential role of red cell transfusion. *Crit Care Med* 33, 1191–1198.

Haas, B. J., Gevers, D., Earl, A. M., Feldgarden, M., Ward, D. V., Giannoukos, G., Ciulla, D., Tabbaa, D., Highlander, S. K., Sodergren, E., et al. (2011). Chimeric 16S rRNA sequence formation and detection in Sanger and 454-pyrosequenced PCR amplicons. *Genome Res* 21, 494–504. doi:10.1101/gr.112730.110.

Hastie, T., Tibshirani, R., and Friedman, J. (2009). *The Elements of Statistical Learning*. New York, NY: Springer New York doi:10.1007/978-0-387-84858-7.

Jain, S., Self, W. H., Wunderink, R. G., Fakhran, S., Balk, R., Bramley, A. M., Reed, C., Grijalva, C. G., Anderson, E. J., Courtney, D. M., et al. (2015). Community-Acquired Pneumonia Requiring Hospitalization among U.S. Adults. *N Engl J Med* 373, 415–427. doi:10.1056/NEJMoa1500245.

Kalanuria, A. A., Ziai, W., and Mirski, M. (2014). Ventilator-associated pneumonia in the ICU. *Crit Care* 18, 208. doi:10.1186/cc13775.

Kalil, A. C., Metersky, M. L., Klompas, M., Muscedere, J., Sweeney, D. A., Palmer, L. B., Napolitano, L. M., O’Grady, N. P., Bartlett, J. G., Carratalà, J., et al. (2016). Executive Summary: Management of Adults With Hospital-acquired and Ventilator-associated Pneumonia: 2016 Clinical Practice Guidelines by the Infectious Diseases Society of America and the American Thoracic Society. *Clin Infect Dis* 63, 575–582. doi:10.1093/cid/ciw504.

Kelly, B. J., Imai, I., Bittinger, K., Laughlin, A., Fuchs, B. D., Bushman, F. D., and Collman, R. G. (2016). Composition and dynamics of the respiratory tract microbiome in intubated patients. *Microbiome* 4, 7. doi:10.1186/s40168-016-0151-8.

Khoury, M., Little, J., and Burke, W. (2003). “ACCE: A Model Process for Evaluating Data on Emerging Genetic Tests,” in *Human Genome Epidemiology: A Scientific Foundation for Using Genetic Information to Improve Health and Prevent Disease*, 217–233.

Kitsios, G. D., Morowitz, M. J., Dickson, R. P., Huffnagle, G. B., McVerry, B. J., and Morris, A. (2017). Dysbiosis in the intensive care unit: Microbiome science coming to the bedside. *J Crit Care* 38, 84–91. doi:10.1016/j.jcrc.2016.09.029.

McKay, H. S., Margolick, J. B., Martínez-Maza, O., Lopez, J., Phair, J., Rappocciolo, G., Denny, T. N., Magpantay, L. I., Jacobson, L. P., and Bream, J. H. (2017). Multiplex assay reliability and long-term intra-individual variation of serologic inflammatory biomarkers. *Cytokine* 90, 185–192. doi:10.1016/j.cyto.2016.09.018.

Mehta, R. L., Kellum, J. A., Shah, S. V., Molitoris, B. A., Ronco, C., Warnock, D. G., Levin, A., and Acute Kidney Injury Network (2007). Acute Kidney Injury Network: report of an initiative to improve outcomes in acute kidney injury. *Crit Care* 11, R31. doi:10.1186/cc5713.

Morgulis, A., Gertz, E. M., Schäffer, A. A., and Agarwala, R. (2006). A fast and symmetric DUST implementation to mask low-complexity DNA sequences. *J Comput Biol* 13, 1028–1040. doi:10.1089/cmb.2006.13.1028.

Morris, A., Beck, J. M., Schloss, P. D., Campbell, T. B., Crothers, K., Curtis, J. L., Flores, S. C., Fontenot, A. P., Ghedin, E., Huang, L., et al. (2013). Comparison of the respiratory microbiome in healthy nonsmokers and smokers. *Am J Respir Crit Care Med* 187, 1067–1075. doi:10.1164/rccm.201210-1913OC.

Narvaez-Rivera, R. M., Rendon, A., Salinas-Carmona, M. C., and Rosas-Taraco, A. G. (2012). Soluble RAGE as a severity marker in community acquired pneumonia associated sepsis. *BMC Infect Dis* 12, 15. doi:10.1186/1471-2334-12-15.

Neto, A. S., Barbas, C. S. V., Simonis, F. D., Artigas-Raventós, A., Canet, J., Determann, R. M., Anstey, J., Hedenstierna, G., Hemmes, S. N. T., Hermans, G., et al. (2016). Epidemiological characteristics, practice of ventilation, and clinical outcome in patients at risk of acute respiratory distress syndrome in intensive care units from 16 countries (PRoVENT): an international, multicentre, prospective study. *Lancet Respir. Med.* 4, 882–893. doi:10.1016/S2213-2600(16)30305-8.

Panzer, A. R., Lynch, S. V., Langelier, C., Christie, J. D., McCauley, K., Nelson, M., Cheung, C. K., Benowitz, N. L., Cohen, M. J., and Calfee, C. S. (2017). Lung microbiota is related to smoking status and to development of ARDS in critically ill trauma patients. *Am J Respir Crit Care Med*. doi:10.1164/rccm.201702-0441OC.

Pattern Recognition and Machine Learning | Christopher Bishop | Springer Available at: http://www.springer.com/us/book/9780387310732 [Accessed December 1, 2017].

R Foundation for Statistical Computing, R. C. T. (2016). *R: A Language and Environment for Statistical Computing*. Vienna, Austria: CRAN.

Schloss, P. D., Westcott, S. L., Ryabin, T., Hall, J. R., Hartmann, M., Hollister, E. B., Lesniewski, R. A., Oakley, B. B., Parks, D. H., Robinson, C. J., et al. (2009). Introducing mothur: open-source, platform-independent, community-supported software for describing and comparing microbial communities. *Appl Environ Microbiol* 75, 7537–7541. doi:10.1128/AEM.01541-09.

Sedgewick, A. J., Shi, I., Donovan, R. M., and Benos, P. V. (2016). Learning mixed graphical models with separate sparsity parameters and stability-based model selection. *BMC Bioinformatics* 17 Suppl 5, 175. doi:10.1186/s12859-016-1039-0.

Segal, L. N., Clemente, J. C., Tsay, J.-C. J., Koralov, S. B., Keller, B. C., Wu, B. G., Li, Y., Shen, N., Ghedin, E., Morris, A., et al. (2016). Enrichment of the lung microbiome with oral taxa is associated with lung inflammation of a Th17 phenotype. *Nat. Microbiol.* 1, 16031. doi:10.1038/nmicrobiol.2016.31.

Singer, M., Deutschman, C. S., Seymour, C. W., Shankar-Hari, M., Annane, D., Bauer, M., Bellomo, R., Bernard, G. R., Chiche, J.-D., Coopersmith, C. M., et al. (2016). The Third International Consensus Definitions for Sepsis and Septic Shock (Sepsis-3). *JAMA* 315, 801–810. doi:10.1001/jama.2016.0287.

Srinivasan, R., Karaoz, U., Volegova, M., MacKichan, J., Kato-Maeda, M., Miller, S., Nadarajan, R., Brodie, E. L., and Lynch, S. V. (2015). Use of 16S rRNA gene for identification of a broad range of clinically relevant bacterial pathogens. *PLoS ONE* 10, e0117617. doi:10.1371/journal.pone.0117617.

Zumla, A., Tawfiq, J. A. Al-, Enne, V. I., Kidd, M., Drosten, C., Breuer, J., Muller, M. A., Hui, D., Maeurer, M., Bates, M., et al. (2014). Rapid point of care diagnostic tests for viral and bacterial respiratory tract infections--needs, advances, and future prospects. *Lancet Infect Dis* 14, 1123–1135. doi:10.1016/S1473-3099(14)70827-8.
